# Cross-species gene-family fluctuations reveal the dynamics of horizontal transfers

**DOI:** 10.1093/nar/gku378

**Published:** 2014-05-14

**Authors:** Jacopo Grilli, Mariacristina Romano, Federico Bassetti, Marco Cosentino Lagomarsino

**Affiliations:** 1 Dipartimento di Fisica e Astronomia “G. Galilei”, Università di Padova, Via Marzolo 8, I-35131 Padova, Italy; 2 Dipartimento di Fisica, Università degli Studi di Milano, via Celoria, 16, 20133 Milano, Italy; 3 Università di Pavia, Dipartimento di Matematica, via Ferrata 1, 27100 Pavia, Italy; 4 CNRS, UMR 7238, Paris, France; 5 Sorbonne Universités, UPMC Université Paris 06, UMR 7238 Computational and Quantitative Biology, Genomic Physics Group, 15 rue de l’École de Médecine, Paris, France

## Abstract

Prokaryotes vary their protein repertoire mainly through horizontal transfer and gene loss. To elucidate the links between these processes and the cross-species gene-family statistics, we perform a large-scale data analysis of the cross-species variability of gene-family abundance (the number of members of the family found on a given genome). We find that abundance fluctuations are related to the rate of horizontal transfers. This is rationalized by a minimal theoretical model, which predicts this link. The families that are not captured by the model show abundance profiles that are markedly peaked around a mean value, possibly because of specific abundance selection. Based on these results, we define an abundance variability index that captures a family's evolutionary behavior (and thus some of its relevant functional properties) purely based on its cross-species abundance fluctuations. Analysis and model, combined, show a quantitative link between cross-species family abundance statistics and horizontal transfer dynamics, which can be used to analyze genome ‘flux’. Groups of families with different values of the abundance variability index correspond to genome sub-parts having different plasticity in terms of the level of horizontal exchange allowed by natural selection.

## INTRODUCTION

While the traditional view of protein evolution focused solely on amino-acid changes, it is now clear that at a higher level of complexity, the proteome evolves using protein domains as elementary structural and functional modules. These building blocks are combined and arranged over evolutionary times, giving rise to gene families (1–3). In bacteria, the modular expansion and contraction of protein families generates highly variable gene repertoires (4,5). Expansion of families is associated with the acquisition of novel functions and novel regulatory structures. Focusing on the protein/domain families found on the same genome, it is well known that they follow a universal distribution with a cutoff correlated with the genome size (6), which is one among other simple but remarkable statistical regularities (7,8). This particular law is essentially due to the interplay of family-expansion and innovation moves (9–11).

On the other hand, the cross-genomic family abundance statistics is comparatively poorly characterized, and is the subject of our attention here. Families expand predominantly by horizontal gene transfer (HGT) or internal gene duplication. HGT may add radically new genetic information, and liberate genomic evolutionary processes from tinkering exclusively with pre-existing genes (4,5,12). Horizontal transfer is believed to be the dominant component of genome innovation for bacteria (13). Cross-, and intra-species HGT are believed to occur at very high rates, leading to very large pan-genomes (14). Conversely, duplicated genes are fixed by processes of sub- or neo-functionalization, which render both copies adaptive. Ample evidence shows that duplication has an important role in bacterial adaptation, for example in genome adaptive expansion (15). Detected duplications in the *E. coli* clade appear to be recent, and preferentially associated to highly expressed genes (13).

Followed across genomes, the gene content variability is often summarized as a ‘gene frequency distribution’, which describes, within a set of sequenced genomes, how many single-copy orthologous genes are found to occur in a given fraction of the repertoired genomes (16–20). The gene frequency distribution contains relevant information about the evolutionary mechanisms that shape the gene content of a species’ genome, but focuses solely on average occurrence, rather than on the variability across genomes. Here, we approach the statistics for cross-species gene-content variation in terms of gene-family abundance.

In this case, besides the presence/absence of a family, one has to deal with the inter-species variability of the number of members of that family (which we term here ‘abundance’) across sequenced genomes. In other words, each family is characterized by a specific ‘fluctuation’ pattern of its abundance across genome rather than by a single mean observable as in the case of gene frequency. These cross-genomic gene-family histograms, or ‘gene-family abundance distributions’, are shaped by events of gene duplication, loss and horizontal transfer.

Using data concerning domain (super)families for 1065 sequenced distinct bacterial species, we find a link between the amount of horizontal transfers within a family and the width of its abundance histogram. We use a minimal stochastic model to rationalize this result. The model predicts an increased dispersion in family abundance as intra-species family expansion (comprising duplication and intra-species horizontal transfer) becomes more relevant over inter-species horizontal transfer. Additionally, we find that a third class of gene families exists, characterized by a family abundance histogram that is more sharply peaked than possibly expected by the model. Analyzing the functional categories of the families that follow this trend, we argue how they could be subject to selection relative to gene abundance. These findings allow us to define an index able to classify some evolutionary properties of gene families purely based on their cross-species abundance histograms, and different genome sub-parts that behave differently in terms of the levels of horizontal exchange and duplication allowed by natural selection.

## MATERIALS AND METHODS

### A collisional model for cross-species gene-family evolutionary dynamics

In the model, a fixed number *N* of species’ genomes interact pairwise randomly, and can gain genes by ‘collisions’ associated with HGT events; the collisions are assumed to be homogeneous, but we will see that this assumption can be relaxed with no effect on the predictions. At each time step, two species are chosen randomly and they can exchange genes by HGT by drawing them from each other's genome with Bernoulli trials of probability *p*_*h*_. During the same time, they draw in a similar way from their own genome a set of genes to be lost (with probability *p*_*l*_) and duplicated (with probability *p*_*d*_). The mathematical formalization of this process is described in the Supplementary Data. Hence, a time step ideally represents the characteristic time between two fixed inter-species horizontal transfer events. Draws from different families are assumed to be independent (see the main text and Supplementary text). To obtain stationarity it is technically necessary to assume that *p*_*h*_ + *p*_*d*_ = *p*_*l*_ (in addition to *p*_*d*_ + *p*_*l*_ ≤ 1), and thus the total number of elements $\sum _{i=1}^N V_i$$\sum _{i=1}^N V_i$ of all families across genomes is, on average, conserved (see Supplementary Text). The model was accessed through both direct simulations and mean-field analytical calculations.

### Data sources

The domain compositions of the proteins of all analyzed bacterial genomes and the superfamily functional annotations were retrieved from the SUPERFAMILY database (21) (release 1.75 27/1/2013), which contains protein domain superfamily assignments using the SCOP (22) structural classification of proteins, for all protein sequences in completed genomes. We considered the 1065 bacterial genome species in the data set, and excluded the strains, as their presence would bias the abundance profiles. We also analyzed separately a set of 225 unicellular eukaryotes merged with data from 62 higher eukaryotes for which the domain assignment of the longest transcript per gene (excluding splicing variants) were available from the SUPERFAMILY database. For the analysis of functional annotations, we considered the function annotation of SCOP domain superfamilies, which is a scheme of 50 detailed functional categories, mapped to seven more general function categories, developed by C. Vogel (23).

Horizontal transfers inferred by the following three independent methods were considered. (i) The Horizontal Gene Transfer Database (HGT-DB (24)), which scores genes that differ statistically from the rest of the genome in G+C content and/or amino-acid usage. (ii) The DarkHorse database (25), which scores phylogenetically atypical genes on a genome assigning a ‘lineage probability index’ (LPI); we considered the set genes having scores higher than the threshold LPI > 0.6 and excluded matches with reciprocal LPI scores <0.75. (iii) The data set from (13), which detected, using phylogenetic methods, gene-family expansion by both horizontal transfer and duplication. For each data set, the analysis was restricted to the set of genes present in the SUPERFAMILY database. The final data set (i) contains a total of 549 genomes and 51 658 domains, data set (ii) a total of 348 genomes and 21 004 domains, and data set (iii) a total of 20 genomes and 1864 domains. Transferred (or duplicated) domains were defined as the assigned SUPERFAMILY domains of a transferred (duplicated) gene. Plasmid family enrichment was computed using the domain assignments of the NCBI plasmid sequence collection available within the SUPERFAMILY database, and the ‘unusual families’ tool of the database web site.

### Data analysis

Genomes were divided into sliding bins based on their size in assigned domains. Each bin contained a size interval of 390 domains; for this size interval, we verified that the mean and the variance of domain superfamily abundance was stable when sliding the bins. The sampling weight *w*_*f*,*b*_ of a (super)family *f*, for each bin *b*, is defined considering the number of genomes in the bin *n*_*b*_ and the number of nonzero entries $n^+_b$$n^+_b$ of the family in the bin, $w_{f,b} = n_b n^+_b/(\sum _b n_b^2)$$w_{f,b} = n_b n^+_b/(\sum _b n_b^2)$. Each family is assigned weight *w*_*f*_ = ∑_*b*_*w*_*f*,*b*_. To calibrate the analysis, we performed a preliminary survey of the abundance histograms for metabolic domain superfamilies. We found that of 318 families, 66 showed abundance distributions that were very similar to Poisson. The rest showed different behaviors. We used this result to define a ‘noise threshold’, to identify the empirical histograms that are sampled sufficiently well, as the value of the sampling weight *w*_*f*_ for the 66th-ranking family (*w*_*f*_ ≃ 0.38). The order parameter *L*_*f*_ is defined as $L_f = \sum _b L_{f,b} n^+_b/(\sum _b n^+_b)$$L_f = \sum _b L_{f,b} n^+_b/(\sum _b n^+_b)$, where, for each bin, the *L*_*f*,*b*_ is the L1 distance of the family abundance histogram in the bin *p*_*f*,*b*_(*v*) with a Poisson distribution Poiss(*v*), $L_{1,f} = \frac{1}{2} \sum _v \left|p_{f,b}(v) - {\rm Poiss}(v) \right|$$L_{1,f} = \frac{1}{2} \sum _v \left|p_{f,b}(v) - {\rm Poiss}(v) \right|$. Sums over *v* were truncated at *v* = 450. The order parameter *Q*_*f*_ is defined as *Q*_*f*_ = ∑_*b*_*Q*_*f*, *b*_*w*_*f*,*b*_, where

\begin{equation*} \begin{split} Q_{f,b} &= (1-\delta _{\mathrm{Var}_b(v_f),0}) \log { \frac{ \langle v_f \rangle _{b}}{\mathrm{Var}_{b}(v_f)}} \ + \\ &+ \ \ \delta _{\mathrm{Var}_{b}(v_f),0} \left( \max _{b}{\log {\frac{\langle v_f \rangle _{b}}{\mathrm{Var}_{b}(v_f)}}} \right). \end{split} \end{equation*}

In the above expression, δ_*k*,*l*_ is Kronecker's delta, *Var* stands for variance, and 〈 》 stands for the mean. *Q*_*f*,*b*_ evaluates the deviation from Poisson behavior in terms of (log) mean-to-variance ratio (inverse Fano factor), considering separately the case of zero variance, which occurs in empirical data. For the analysis of horizontal transfers, the parameter *H*_*f*_ was defined as a mean fraction of transfers over the family size as follows:

\begin{equation*} H_f = \frac{1}{O_f} \sum _g \frac{H_{f,g}}{V_{f,g}}, \end{equation*}

where the index *g* = 1. *O*_*f*_ runs on genomes where the family *f* is present, *H*_*f*,*g*_ is the number of scored horizontal transfers for family *f* in genome *g*, and *V*_*f*,*g*_ the abundance of family *f* in genome *g*.

## RESULTS

### Model simulations and calculations

**Figure 1.**
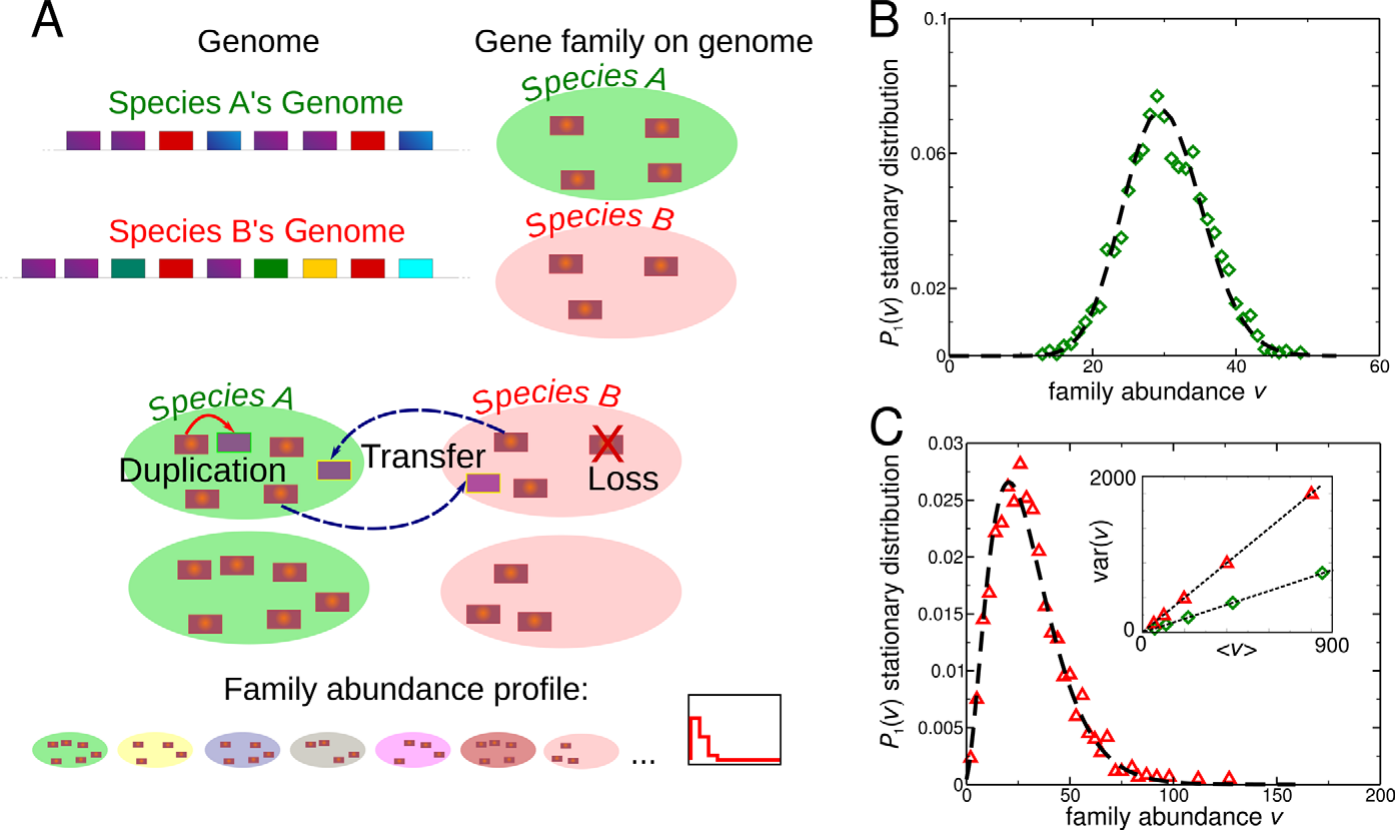
Definition and main predictions of the model. (**A**) Sketch of the model. For each genome representative of a species (top), the model describes the dynamics of each gene family separately over a set of species, represented here (for each species) as the Venn diagram of the elements of the family. Following stochastic binary ‘collision’ events between species (middle), genes of a given family are exchanged between species, and, over the same time scale, can be duplicated or lost in each species. (**B**) In absence of gene duplications, the model predicts a Poisson distribution for the family abundance profile. Symbols (green diamonds) are the steady-state abundance histogram from simulation of 1000 species, with *p*_*d*_ = 0 *p*_*h*_ = 0.01 and initial abundance of 30 for all species. The dashed line is the analytical prediction (a Poisson distribution with average 30). (**C**) In presence of duplications, the dispersion of the steady-state abundance histogram increases. Symbols (red triangles) represent the steady-state abundance histogram from simulation of 1000 species, with *p*_*d*_ = 0.009 *p*_*h*_ = 0.001 and initial abundance of 30 for all species. The dashed line is the analytical prediction, a negative binomial distribution, valid in the limit of small *p*_*d*_ and *p*_*h*_ (see text). The inset shows that in both cases (same symbols as above) the analytical estimates (dashed lines) capture well the scaling of the variance of the abundance profile with the average abundance found in simulations (symbols).

We discuss first the stochastic model (Figure 1A), since the results are useful to introduce the data analysis. The model describes a minimal dynamics of duplication/loss and inter-species HGT, and formulates a minimal informed expectation for the family abundance profile. The model only describes events that are visible on the representative genome of the species (because they are fixed), and recapitulates the action of selection in the rates *p*_*d*_, *p*_*h*_ and *p*_*l*_. Importantly, when compared to data, the model only describes inter-species events, and thus the ‘duplication’ move is an intra-species family expansion that includes duplication as well as intra-species horizontal transfers. For simplicity, we will mainly refer to the move as duplication in the description of the model, and explicitly address the question when dealing with the data. Finally, we assume independence between gene families. Thanks to the latter condition, the gene abundance *V*_*i*_ of a single family across all *i* = 1...*N* species can be described separately from the others. Note however that, while matching the model with empirical data, the effective rates are allowed to vary from family to family, giving rise to the observed diversity between families, hence this simplifying assumption is not restrictive. Model time maps to evolutionary time in a complex way. In comparing with data, we will assume that observed species had the time to reach a steady state where the gene-family abundance distributions are roughly invariant (i.e. that the stationary abundance distribution is the empirically relevant quantity). The main observable is the family abundance profile, the distribution of the family population *V*. Using mean-field kinetic equations similar to Boltzmann equations (26), it is possible to estimate the stationary-state value of all moments of *V*. Processes of the type considered have already been applied in various interdisciplinary contexts (27–30).

### In presence of horizontal transfer and loss the model predicts a stationary Poisson distribution

Setting *p*_*d*_ = 0, the only effective parameter is *p*_*h*_ = *p*_*l*_. In this regime, the dynamics of a family is dominated by horizontal transfer and loss. Since in the simulated dynamics HGTs are balanced with losses, the mean abundance λ is fixed by the initial conditions. Assuming that these averages are determined by extrinsic or ancestral processes, we ask which kind of abundance profile can be maintained in the hypothetical scenario of ‘steady’ evolution where duplications (and intra-species HGT gains) are irrelevant. Simulations show that, starting from an arbitrary initial condition, the system relaxes to a stationary state where the family abundance profiles *P*(*V* = *v*) do not vary with time. The stationary abundance histogram resembles a Poisson distribution with parameter λ (Figure 1B). Importantly, when *p*_*d*_ = 0, we prove analytically that the mean-field steady*-*state solution is the Poisson distribution of parameter λ (see Supplementary Text). Note that this prediction is parameter-free, as the only parameter of the Poisson distribution is fixed by the average (Figure 1B).

### When duplication is relevant the model predicts overdispersed stationary distributions

When *p*_*d*_ > 0 and duplication (or intra-species transfers) cannot be neglected, all three parameters are relevant to define the steady state. Simulations show that increasing *p*_*d*_ makes the steady-state abundance distribution increasingly dispersed (Figure 1C), as confirmed by analytical mean-field calculation of the moments of the distribution (see Supplementary Text). At steady state, we obtain Var(*V*) = λ(1 + *p*_*d*_/(*p*_*h*_(1 − *p*_*h*_))), which perfectly agrees with simulations (Figure 1C). A closed analytical expression for the full distribution can be obtained in the so-called ‘grazing collisions’ limit (31), i.e. when *p*_*d*_ → 0 and *p*_*h*_ → 0 with the same rate, and is a negative binomial distribution of mean λ and variance λ(*p*_*d*_ + *p*_*h*_)/*p*_*h*_ (see Supplementary Text).

The relaxation times of all moments of *V* can be estimated analytically in the mean-field limit (Supplementary Figure S1). In general, the steady state is approached exponentially with relaxation time 1/(*p*_*h*_(1 − *p*_*h*_)), measured in ‘sweeps’ of *N* collisions (see Supplementary Text and Figure S1). This result agrees well with finite-*N* simulations. As we mentioned, model time has a complex relationship with evolutionary time. However, the relevant aspect is that a certain number of fixed horizontal exchanges are required to build up the steady distribution (the relaxation time depends only on *p*_*h*_, even when *p*_*d*_ > 0).

### The range of behaviors predicted by the model is found in empirical data

The model contains a number of radical assumptions, but guides the analysis of the empirical gene-family abundance distributions. As we will see, the distributions predicted by the model are present in a relevant fraction of the empirical families and correspond very well to their expected biological and evolutionary features.

We have considered data of domain assignments relative to 1065 sequenced bacteria of different species from the SUPERFAMILY database, which provides a coarse-grained definition of a domain family/superfamily, and thus allows to obtain well-sampled per-family abundances. We performed the main analyses on a total of 1530 superfamilies, and verified that the results were consistent at the family taxonomy level. For each superfamily (or family), indexed by *f*, we computed the abundance profiles *P*^*f*^(*v*). Note that this approach is complementary to the customary approach of phylogenetics studies, where the main focus are presence–absence patterns of orthologs. The abundance counts include orthologs, but mostly xenologs (horizontal transfers) and paralogs (duplicates), and thus in principle contains information about the processes (described by the model) through which new genes are added on a genome. Thus, abundance and presence/absence patterns report about very different information.

It is well-known that family abundance is related to genome size (11), and also that the total population of families with specific biological functions has definite scaling with genome size (32–34). Furthermore, the amount of horizontal transfers increases with overall family size (35). Thus, in order to bypass possible spurious signals, we computed family abundance histograms for sets of genomes belonging to (sliding) bins of similar genome size, measured in domains. This procedure can be justified theoretically, using a model variant that accounts for different genome sizes in a simplified way (see Supplementary Text).

Additionally, many families are sparsely (or poorly) populated, and genome size bins of constant width contain a highly variable amount of genomes. In order to compensate for these sampling problems, we used the weights *w*_*f*_, which measures the efficiency of sampling.

**Figure 2.**
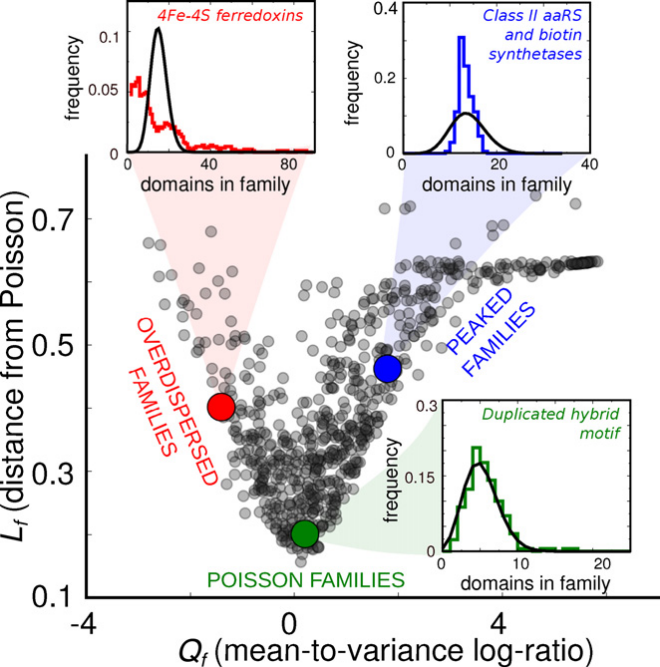
Typical behaviors of the domain (super)family abundance profiles and their classification. Three main behaviors exist for the families, shown by the insets. Each inset compares an example of family abundance histogram (steps) with the Poisson distribution with equal mean (black lines). ‘Poisson-like’ family profiles correspond to this expectation, while ‘overdispersed’ and ‘peaked’ profiles are characterized by larger and lower variance, respectively. The scatter plot represents two different ways to score the deviation from Poisson-like behavior. Each point represents a domain (super)family. The *x*-axis reports the order parameter *Q*_*f*_, which quantifies the deviation from the equality of mean and variance by their log ratio. The *y*-axis is the parameter *L*_*f*_, based on the *L*1 distance between the abundance histogram and the Poisson distribution with equal average. Both observables are weighted on the sampling noise.

A survey of the families with well-sampled abundance profiles (high *w*_*f*_) shows that the model phenomenology is present in the data (Figure 2; some further representative examples are given in Supplementary Figure S2). Specifically, some families have abundance profile histograms that are very close to Poisson distributions, and some others have a higher dispersion. A third type of family abundance profile is not captured by the model, as it is less dispersed than the Poisson distribution with equal average. A few of these families have zero variance (i.e. they are deterministically populated by the same number of domains across genomes).

### Order parameters and behaviors of the gene-family abundance distributions

To further quantify these observations, we have used the statistics, or ‘order parameters’, *L*_*f*_ and *Q*_*f*_. According to the model prediction, a family with null behavior, and whose evolution is dominated by horizontal transfers, should have an abundance histogram that looks like a Poisson distribution, and whose average is equal to the mean. The order parameters *L*_*f*_ and *Q*_*f*_ measure the deviation from these two properties (in particular, *Q*_*f*_ is equivalent to an inverse Fano factor, see the Materials and Methods section). Both *Q*_*f*_ and *L*_*f*_ should be close to zero if the abundance profile complies to the model prediction for the case of negligible duplications, a Poisson distribution. The scatter plot of *L*_*f*_ versus *Q*_*f*_ (Figure 2) shows that this property is valid for a set of families.

The same plot in Figure 2 allows to distinguish families based on their abundance profiles: in the data, abundance profiles deviate from Poisson behavior in two ways: by increasing their variance (i.e. going to negative *Q*_*f*_) or by decreasing it (positive *Q*_*f*_). The families that increase it, also increase their dispersion, and are, in principle, captured by the model variant that includes duplication. The families whose variance in the abundance profile is decreased escape the null model, suggesting that they might be subject to further constraints limiting their abundance fluctuations.

**Figure 3.**
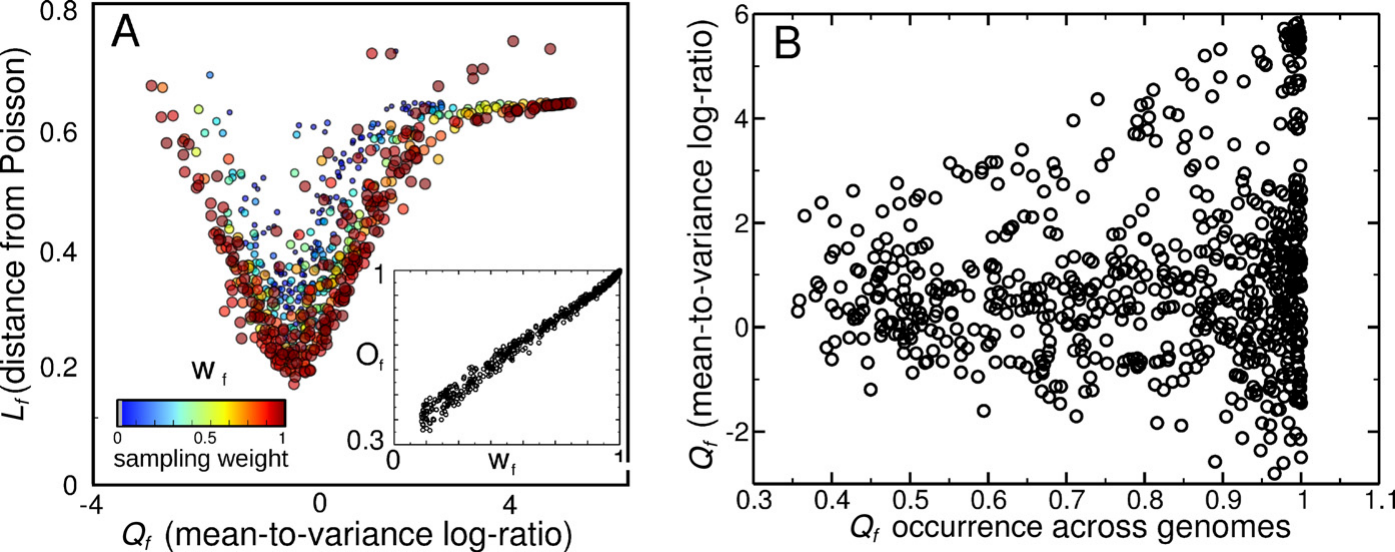
Robustness of the abundance profile classification with respect to the sampling weight of families *w*_*f*_*.* (**A**) The scatter plot is the same as the one in Figure 2 of the main text, but color and size of each point correspond to the sampling weight *w*_*f*_. The ‘V’ shape of the relation between *L*_*f*_ and *Q*_*f*_ is more marked for the families with high *w*_*f*_. Inset: linear relation between sampling weight *w*_*f*_ and the occurrence *O*_*f*_ of families. The latter is defined as the fraction of genomes having at least one domain of the family considered. The fact that high *w*_*f*_ families are not preferentially associated with any value of *Q*_*f*_ indicates that the classification operated by *Q*_*f*_ is robust. (**B**) Lack of correlation between *O*_*f*_ and *Q*_*f.*_

The families with lower sampling noise (high *w*_*f*_) tend to have lower *L*_*f*_ at equal *Q*_*f*_ and to collapse around a common *L*_*f*_(*Q*_*f*_) curve (Figure 3A). In particular, for *Q*_*f*_ ≃ 0 their abundance profiles resemble more, and more consistently across genome sizes, a Poisson distribution. The collapse of the data with increasing sampling efficiency can be considered an important consistency check with the model, which predicts common underlying abundance profiles. We also found that the sampling weight *w*_*f*_ linearly correlates very strongly with the family occurrence, i.e. the fraction of genomes where family *f* is found (Figure 3A). The occurrence is the domain family equivalent of the so-called ‘gene frequency’ of orthologs (16–20), and follows a similar distribution. Conversely, the abundance fluctuation index *Q*_*f*_ does not show any clear correlation with the occurrence, indicating that in the data abundance fluctuations and occurrence are distinct (Figure 3B). In addition, the analysis is very robust with respect to domain taxonomy (SCOP families versus superfamilies, Supplementary Figure S3).

Importantly, the fluctuation index *Q*_*f*_ is consistent for groups of genomes with different size (Supplementary Figure S4), while the abundances typically scale with size. In other words, with varying genome size, the index *Q*_*f*_ robustly classifies a scaling of the variance with the mean, which seems to be a family-specific feature. This supports the conclusion that family abundance fluctuations essentially follow three main trends, which are quantified by the index *Q*_*f*_: ‘Poisson-like’ abundance profiles tend to follow a Poisson distribution, while ‘overdispersed’ and ‘peaked’ profiles have, respectively, increased and decreased species-to-species variability. Notably, the same classification is less consistent for abundance fluctuations in eukaryotic genomes (Supplementary Figure S5), where horizontal transfers are much rarer, and the underlying picture suggested by the model is not expected to readily apply.

The above analysis indicates that, assuming the model, the cases *p*_*d*_ = 0 and *p*_*d*_ > 0 should be well distinguished by *Q*_*f*_, and that the distributions are Poisson-like or not exactly according to the model prediction following the values of this parameter. However, we did not attempt any precise parameter inference. The model has three parameters, and the size conservation assumption reduces them to two. However, if *p*_*d*_ = 0, the value of *p*_*h*_ cannot be easily inferred because *Q*_*f*_ is insensitive to this parameter. Additionally, in the grazing collision limit, the only recognizable parameter is *p*_*d*_/*p*_*h*_. In general, additional knowledge of the skewness of the abundance profiles should in principle allow to estimate *p*_*d*_, but in the empirical data the histograms appear too noisy to perform this operation reliably.

### Evolutionary behavior and gene-family abundance profile

The model suggests that different values of *Q*_*f*_ should also correspond to different rates of cross-species HGTs and duplications/intra-species HGTs. To obtain a direct proof that abundance fluctuations are connected with rates of fixed HGTs and gene duplications, we employed four bioinformatics data sets that measured these quantities on a large set of genomes, and compared the amounts of scored transfers and duplication with the index *Q*_*f*_. We converted the gene-based data into domains using the simple criterion that all domains belonging to transferred or duplicate genes are scored as transferred or duplicate domains.

**Figure 4.**
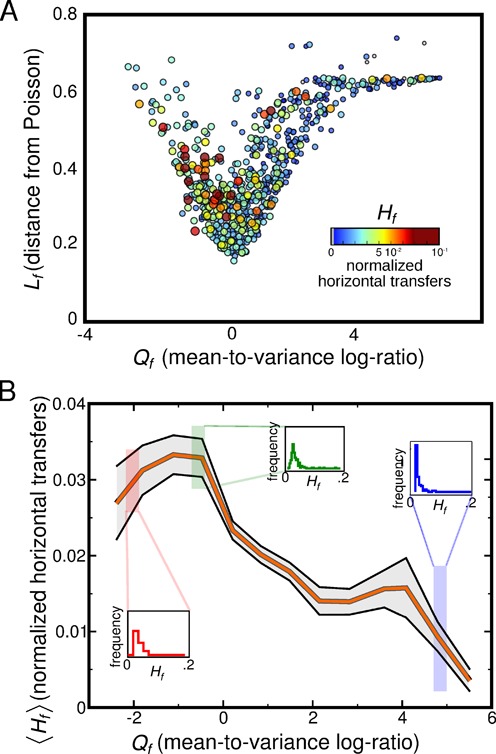
Independent estimates of horizontal transfers within a family correspond to the model expectation from the family abundance profiles. (**A**) The scatter plot is the same as the plot in Figure 2, but the color and size of each point correspond to the parameter *H*_*f*_, which is an average number of horizontally transferred domains in that family (estimated here using data from the HGT-DB database), normalized, for each genome where family *f* is found, to the family size. Points with *H*_*f*_ = 0 are in gray. Compatibly with the expectation of the model, many horizontal transfers are found for Poisson-like families, i.e. toward the minimum of this plot. For *Q*_*f*_ > 0 (peaked) families tend to have high or null *H*_*f*_. (**B**) Plot of the average of *H*_*f*_ over classes for bins of *Q*_*f*_. *H*_*f*_ increases with decreasing *Q*_*f*_, indicating that an increasing number of transfer events are found for families with Poisson-like and overdispersed abundance profiles, compatibly with the null model expectations. The insets show the histograms of *H*_*f*_ for the region connected to them. See Supplementary Figures S6 and S7 for related tests using different data sets.

The first data set, HGT-DB (24), scores putative horizontal transfers on a set of 959 species’ genomes using GC content. For each family and genome, this data source estimates the putative genes, and hence domains, gained by transfers, which can be subdivided into families. Each family yields a certain number of transfers per genome. For each family and genome, we normalized the number of horizontally transferred domains to the family size. We defined the parameter *H*_*f*_ of a given family as the average across genomes of this quantity. The cross-genomic average compensates in part the obviously larger sampling errors for families with lower abundance. *H*_*f*_ quantifies the relative contribution of HGT to a family; it approaches 1 when the family has many putative transfers consistently across genomes, and 0 when very few transfers are typically found compared to the family size. Figure 4 shows that families with Poisson-like and overdispersed abundance profiles (*Q*_*f*_ ≲ 0) tend to have higher *H*_*f*_, and hence carry more horizontal transfers. This is in line with the model predictions (considering that intra-species transfers and duplications are not distinguished by the model). Additionally, we repeated the analysis considering data from the DarkHorse database (25), which scores horizontal transfers on genome-wide basis by a complementary approach, based on phylogenetic information from the NCBI taxonomy database (36), and the results are very robust (Supplementary Figure S6).

**Figure 5.**
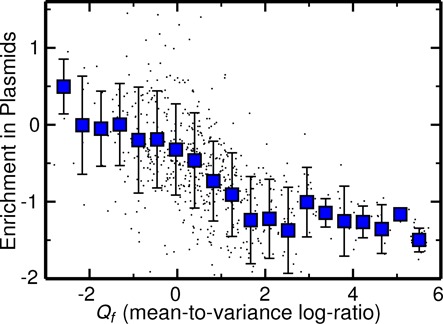
Enrichment of dispersed families on mobile elements (plasmids). The abundances of domain superfamilies on plasmids were obtained from domain assignments of the NCBI plasmid sequence collection. The plot shows the correlation of *Q*_*f*_ with enrichment of families on plasmids. The *y*-axis represents a log ratio score (21) between the percentage of total plasmid domains belonging to a given superfamily and the background percentage computed on all bacterial genomes. This score increases for families that are increasingly enriched on plasmids.

Third, we tested the correlation of *Q*_*f*_ with the enrichment of a family on plasmids as a proxy for the tendency of the family members to be found on ‘mobile elements’. To perform this analysis, we used the domain assignments of the NCBI plasmid sequence collection. We found that families with *Q*_*f*_ ≲ 0 tend to be more enriched on plasmids, while families with increasingly larger positive values of *Q*_*f*_ are increasingly underrepresented (Figure 5), in line with the idea that abundance fluctuations are related to family ‘mobility’ across genomes.

To assess the role of transfer and duplication in gene-family expansion, we analyzed the data set from Treangen and Rocha (13), who estimated the relative contributions of horizontal transfer and duplication to gene-family expansion in a set of closely related complete genomes. In this case, we found results that are consistent with the above conclusions, but the small size of the data set did not allow a thorough statistical analysis (Supplementary Figure S7). In particular, the families that were scored as containing abundant gene duplications consistently have *Q*_*f*_ < 0, as expected from the null model.

Finally, in order to further test whether families with different abundance variability build up genome sectors with differential plasticity, we defined three pairwise genome distances based on superfamily usage (37) restricted to families with overdispersed, Poisson-like and peaked abundance profiles, respectively. For each genome pair in the data set, we thus obtained three distances, which we compared to a reference distance obtained from a 16S ribosomal RNA phylogenetic tree (38). While family-based metrics are usually a good signal of phylogeny, HGTs should ‘scramble’ the signal from vertical descent. In line with this, the genome distances based on Poisson-like and overdispersed family content are less correlated with phylogenetic distance (Spearman ρ 0.36 and 0.32, respectively) compared to the distance based on peaked families (Spearman ρ 0.46).

### Biological function and gene-family abundance profile

As well known, the evolutionary properties of a gene family are connected to its functional landscape. Thus, if the classification of gene families based on their abundance histograms is meaningful, one expects a connection between biological function and abundance profiles of the families. We performed Fisher exact tests on the contingency of different functional categories and abundance profile classes (Table 1). To avoid biases from undersampled families, we restricted the analysis to the subset of 701 (super)families that fall below a ‘noise threshold’ value of *w*_*f*_. In order to perform the analysis, we divided these families according to their abundance fluctuations into the following three classes: ‘overdispersed’ (*Q*_*f*_ < −0.43, 130 families), ‘peaked’ (*Q*_*f*_ > 1, 281 families) ‘Poisson-like’ (−0.43 < *Q*_*f*_ < 1, 282 families).

**Table 1.** Relation of the abundance profile of a family with its biological function

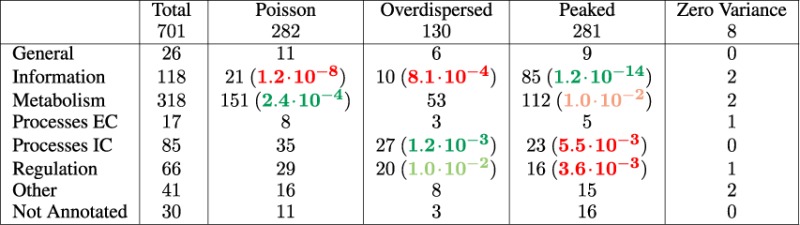					

The counts for the larger functional categories of domain superfamilies divided according to their abundance profile histograms following thresholds on *Q_f_* (see text) are reported. *P*-values for Fisher's exact tests are reported in parenthesis when significant (*P* < 0.01), in green for overrepresentation and in red for underrepresentation. Only the 701 families below the noise threshold for the family abundance histograms (see text) are considered. A wider classification yields similar results (Supplementary Table S1). A similar analysis with the finer functional categories is presented in Supplementary Table S2.

We used the SCOP superfamily domain functional annotations, which comprise 50 detailed functional categories, mapped to seven broader categories: information, regulation, metabolism, intracellular processes, extracellular processes, general, other/unknown. The associations with these broader functional categories are in line with previous reports and with general knowledge on horizontal transfer/duplication dynamics in bacteria (4,5,39). Metabolic genes are significantly enriched with Poisson-like abundance profiles, and in negative association with peaked families. This supports the idea that these domain (super)families are mainly sculpted by horizontal transfers. The gene-regulation category is significantly associated with overdispersed families, and underrepresented in peaked families, and intracellular processes follow the same trend. Finally, the information functional category is significantly associated with peaked families, and strongly underrepresented in overdispersed and Poisson families, suggesting that families related to translation should be subject to abundance constraints, absent in the null model. A parallel analysis that includes the noisy classes yields results that are very consistent (Supplementary Table S1).

The analysis of the finer functional categories is presented in Supplementary Table S2. Although affected by small set statistics, this analysis contains some further information. As expected, most of the signal from the information functional category comes from families associated with translation. For metabolism-associated families, the tendency to have a Poisson-like abundance histogram is particularly strong for domain topologies associated with oxidation/reduction. In the regulation functional category, DNA-binding/transcription factor families are associated with overdispersed abundance histograms. A similar trend is found for signal transduction (such as, e.g. the GAF or EAL domains), while kinase/phosphatase domain families are more strongly associated with Poisson-like abundance profiles. Within the intracellular processes category, families associated with proteases and transport (e.g. porins) also follow a similar trend, while protein modification (chaperones) is enriched for peaked family abundance profiles. Finally, within the extracellular processes category, cell adhesion families are significantly associated with overdispersed profiles. Interestingly, transposases, which are a main driver of mobile elements in bacterial genome, do not show any specific pattern in abundance fluctuations, and different transposase superfamilies are found to have a wide range of values of the index *Q*_*f*_..

## DISCUSSION

The topic of which families and classes of genes can be more frequently transferred than others has been thoroughly studied in recent years (40–43). This study takes a complementary approach and focuses on the inter-species family abundance statistics, offering a novel observable, the abundance fluctuations, which our analysis connects to family-specific HGT dynamics. Importantly, abundance fluctuations are nontrivially distinct from family occurrence (average presence across species), which is the equivalent for domain families of the gene-frequency distribution (16–20). Indeed, the occurrence of a family is not a good predictor of the abundance fluctuation index *Q*_*f*_ (Figure 3B). Hence, abundance fluctuations are not recapitulated by occurrence, which appears natural from a biological standpoint, as adding or removing an entire family from a genome is different, in terms of accessible functional landscape, than adding or removing single family members.

The null model, which we use as a tool to rationalize the link between abundance and HGT dynamics, is based on the following assumptions. The main (null) assumption is that inter-species gene-family abundance fluctuations correspond, effectively, to a stochastic process that realizes a minimal description of inter-species HGTs, intra-species family expansion (by duplication and HGT), and gene losses occurring in a family (7,44). The model focuses on species and does not include population dynamics (45,46); this approach has proven successful in other contexts (9–11,33,47). Each event occurring in one species is to be interpreted as the fixation for the gain or loss of a gene of a particular family in the species representative genome. A final simplifying assumption describes different gene families as independent random variables. It is likely that the abundance of different gene classes depends on biological interactions between them (32). Here, family interactions are described effectively as family-specific effective rates of fixed events. Differently from other descriptions, where horizontal transfer dynamics is treated as an effective interaction with some background gene pool (11,18,20,33), our minimal model describes cross-species horizontal transfers by explicitly accounting for interactions between genomes. The resulting ‘collisional’ formalism has a parallel with the framework classically used by Boltzmann in statistical physics (26). While this has no deep scientific meaning, it has some technical significance: it allowed us to make use of existing analytical techniques to directly access and control the model (48).

One further observation that should be made is that the model assumption of pairwise random interaction between genomes is rather primitive. The assumption is equivalent to considering the genomes equally related to each other (i.e. no genome-level tree exists). This is a gross simplification that is only partially mitigated in our data analysis by using a species-representative set of genomes. Instead, inter-species HGTs occur with decreasing probability for increasing evolutionary distance between genomes (49). The standard formulation of the model does not include this bias, and assumes equal probability of collisions for all genomes. For this reason, we considered a model variant where the collisions are biased by the phylogenetic distance between species (less probable for increasingly distant species). Our simulations (Supplementary Figure S8) show that the results are robust if a bias in the probability of horizontal exchanges is introduced. We estimated this bias from the phylogenetic distances of empirical genomes in our data set, scored by the occurrence patterns of domain families (37).

We now discuss the functional enrichment of families with different abundance profiles. Three main patterns appear. Poisson-like abundance profiles correspond to families dominated by HGT, enriched for metabolic enzymes and kinases/phophatases. Families with overdispersed abundance profiles are characterized by an interplay of HGT and duplications (or intra-species HGT), and enriched for DNA-binding/transcription factors, signal transduction, and functional categories related intracellular processes such as transport and proteases. Families with peaked abundance profiles typically show no HGTs and are highly enriched for translation-related functions. In more detail, metabolic enzymes typically have Poisson-like abundance profile, and thus should be dominated by HGT with respect to duplications. Transferred metabolic enzymes are reported to outnumber duplicated enzymes in the recent evolution of *E. coli*, determining adaptation to new environments (12). Conversely, transcription factors are associated with overdispersed abundance profiles, which, in the model, correspond to increased gene duplications or intra-species horizontal transfers. Analyses of transcriptional regulatory networks (50,51) have found an enrichment of interactions involving paralogs, which can be explained by regulatory divergence after gene duplication. However, since it is well known that horizontal transfer should be the main drive of bacterial genome innovation, this observation has generated some debate (52,53). A recent observation that might reconcile the debate is that widespread intra-species transfers of regulatory regions could explain the regulatory divergence (54). Since the model focuses on inter-species events, it is compatible with this hypothesis. Indeed, as mentioned above, the model does not distinguish duplications from intra-species transfers. A recent analysis indicates that the amount of transfers is proportional to family size (35); it is presumable that horizontal transfers may effectively follow a rich-gets-richer principle, similarly to gene duplications (55), in line with the model assumptions. Regarding the data analysis, the large-scale data available to us only scored transfers, which are frequent for families with overdispersed abundance profiles. A finer, smaller-scale analysis also including duplications is compatible with our results (Supplementary Figure S7), but does not allow to formulate any specific hypothesis on the relative roles of intra-species transfers and duplications in families with overdispersed abundance profiles.

Families with peaked abundance profiles or zero variance are not captured by the model, and hence falsify its null assumptions. Since their abundance varies less than expected across genomes, we speculate that these families might be subject to adaptive abundance constraints, i.e. the domains are needed only in a certain number of ‘copies’, probably corresponding to properties of the protein machines that they form. Notably, while the majority of the peaked abundance profiles correspond to families where little or no horizontal transfers are reported, there are peaked families with high HGT: we have considered the set of top 15% *H*_*f*_ families for both the HGT-DB and DarkHorse data sets, and we found 37 and 35 peaked families, respectively. These lists are mildly enriched only for the metabolism functional category (21/37, *P* = 0.01 for HGT-DB data and 18/35, *P* = 0.2 for DarkHorse; the full lists are given as supplementary files), while the other categories (including translation, which is enriched in peaked families) appear sparsely. The domains common to both lists include two domains for the metabolic enzyme cysteine methyltransferase, involved in DNA repair, the ligase domain ADC synthase, and a gated mechanosensitive ion channel domain. Notably, five distinct ATP synthase domains are found in the HGT-DB list of peaked families with high *H*_*f*_; this gene has a very unusual phylogenetic distribution indicative of HGT (56). Our observations lead to hypothesize the presence of a (to our knowledge so far unreported) constraint on the abundance, possibly of selective origin. A more detailed investigation of this issue might be worth the effort.

Our analysis indicates that the abundance profile of a family generates an informed hypothesis about its function and evolution. More importantly, this suggests a ‘segmented’, or modular view of the genome (57), where the inter-species diversity of a gene family is highly dependent on its biological function. These considerations are in line with those emerging from pan- and core-genome analyses (58), but considering protein abundance fluctuations gives a different perspective than gene presence/absence patterns. For example, we have shown evidence that gene families on a genome with different abundance variability will also have different plasticity (or ‘fluidity’) (14,59), determined by the rates of the different evolutionary processes. As a result, some gene families will see the phylogenetic tree as a tree, while some others will perceive it as a network (whose effective topology can vary from family to family) (5,60).

## CONCLUSION

The results show that cross-genomic family abundance fluctuations give access to relevant information regarding the processes by which gene families expand and contract in genomes and on the exchange of genes between genomes. We introduced a novel modeling framework to enhance and guide the data analysis. The model can help rationalize the most important finding of this work, which establishes a link between cross-species abundance fluctuations and HGT dynamics. The joint analysis of model and data suggests a classification of families based on their cross-genomic abundance histograms, which is biologically meaningful, as the different types of abundance profiles correspond well with the reasonable expectations in terms of biological functions. Importantly, while the necessarily simplifying model assumptions can be discussed, the resulting classification of the data is fully coherent for genomes with different size ranges and finds direct support from cross-comparisons with independent data sources. This suggests that its validity goes well beyond the necessarily radical simplifications of the model. Finally, we showed how abundance fluctuations provide a new perspective on genome plasticity. We speculate that a more detailed quantification of plasticity variation across families will be important in future studies to assemble a unified evolutionary view of genome architecture.

## SUPPLEMENTARY DATA

Supplementary Data are available at NAR Online.

SUPPLEMENTARY DATA
