# Supplementary material for: Cross-species gene-family fluctuations reveal the dynamics of horizontal transfers
Source: Nucleic Acids Res. 2014 May 14;42(11):6850–60. doi: 10.1093/nar/gku378 (PMC4066789; doi:10.1093/nar/gku378)
Supplement: SUPPLEMENTARY DATA [file supp_42_11_6850__index.html]

Cross-species gene-family fluctuations reveal the dynamics of horizontal transfers — SUPPLEMENTARY DATA 

# Cross-species gene-family fluctuations reveal the dynamics of horizontal transfers

## SUPPLEMENTARY DATA

**Files in this Data Supplement:**

- SUPPLEMENTARY DATA
