# Supplementary material for: Cross-species gene-family fluctuations reveal the dynamics of horizontal transfers
Source: Nucleic Acids Res. 2014 May 14;42(11):6850–60. doi: 10.1093/nar/gku378 (PMC4066789; doi:10.1093/nar/gku378)
Supplement: SUPPLEMENTARY DATA [file supp_gku378_nar-00228-z-2014-File010.pdf]

Supplementary Material for Grilli *et al.*
Cross-species gene family fluctuations reveal the dynamics of horizontal transfers

Jacopo Grilli, Mariacristina Romano, Federico Bassetti, Marco Cosentino Lagomarsino

Supplementary Note on Analytical Solutions of the Model

This sections gives further details on the models and its analytical solution in different limit-cases.

Formulation of the model

The model describes the dynamics of the number of elements of a given family $V_i(\tau)$ within an ensemble of $i = 1, \dots, N$ species, in terms of a fictitious time τ where "collisions" occur, corresponding to (binary) events of (fixed) horizontal transfers. To each transfer event, there corresponds a number of (fixed) gene duplications and gene losses. The model assumes that families are independent, and under this assumption, can deal with a single family at a time without loss of generality. At each time step two randomly chosen genomes i and j interact as follows

$$\begin{aligned} V_i(\tau+1) &= V_i(\tau) + \Lambda_i[V_i(\tau)] + H_j[V_j(\tau)], \\ V_j(\tau+1) &= V_j(\tau) + \Lambda_j[V_j(\tau)] + H_i[V_i(\tau)], \end{aligned} \tag{S1}$$

where the random function Λ represents the duplications and losses, and H represents horizontal transfers (which depend on the size of the family in the "donor" genome, as described by the indices).

We choose to describe the collisions in terms of Multinomial events, i.e. a species draws at random gene family members for losses and duplications (from its own genome), and transfers (from the other species' genome). In other words, $\Lambda[v] = \sum_{k=1}^{v} X_k^*$ and $H[v] = \sum_{k=1}^{v} X_k$, with $X_1^*, X_2^*, \dots, X_l, X_2, \dots$ independent discrete random variables, and

$$\begin{aligned} &P\{X_k^* = -1\} = p_l, \quad P\{X_k^* = 1\} = p_d, \\ &P\{X_k^* = 0\} = 1 - (p_d + p_l) \qquad (k \geq 1); \\ &P\{X_k = 1\} = p_h, \quad P\{X_k = 0\} = 1 - p_h \qquad (k \geq 1). \end{aligned} \tag{S2}$$

If $X_k = -1$, the k-th gene is lost, while if $X_k = +1$ it is duplicated. Analogously, if $X_k = 1$ the k-th gene is transfered from the donor genome. Here, p_d,p_l and p_h are the basic relevant parameters of the model, representing the (relative) rates of gene duplication, loss, and horizontal transfer respectively.

We assume that $p_h + p_d = p_l$ and hence $\langle X_k + X_k^* \rangle = 0$ ($\langle \cdot \rangle$ is the expectation value). Under this condition, the total number of elements is conserved in mean, i.e., $\left\langle \sum_{i=1}^N V_i(\tau) \right\rangle = \left\langle \sum_{i=1}^N V_i(0) \right\rangle$. Note that loss has to dominate over duplication (or be the only possible drive) in order for the condition to be fulfilled. Note also that, since $0 < p_l \leq 1$ and $p_l + p_d \leq 1$, one has the constraint $2p_d + p_h \leq 1$.

The numerical simulation of the model was coded in C++, and typically solved for an initial conditions with equal number of family elements of all the genomes, $V_i(0) = v_0 \quad i = 1, \ldots, N$, until stationarity was reached. The simulation code is available with the authors upon request.

Mean-field equation

As described above, the model deals with the dynamics of the number of elements of a given family $V_i(\tau)$ within an ensemble of $i = 1, \ldots, N$ species, in terms of a fictitious binary collision time τ, corresponding to events of fixed horizontal transfers. To each transfer event, there corresponds a number of fixed gene duplications and gene losses.

The full information about the process at time τ is contained in the N-particle joint probability distribution $P_N(v_1, v_2, \ldots, v_N, \tau)$. In order to approach the problem analytically, one can write a kinetic equation for 1-marginal distribution function $P_1(v, \tau) = \sum_{v_2,\ldots,v_N} P_N(v, v_2, \ldots, v_N, \tau)$ involving only one- and two-particle distribution functions, which generates an infinite hierarchy of equations of BBGKY type. The standard mean-field approximation to the interacting particle system model assumes $P_2(v_i, v_j, \tau) = P_1(v_i, \tau)P_1(v_j, \tau)$. This approximation, rescaling the time as $t = 2\tau/N$ and taking the limit $N \to \infty$, gives the Boltzmann-like kinetic equation

$$\frac{\partial}{\partial t} P_t(v) = Q(P_t, P_t)(v) \tag{S3}$$

where $P_t(v) = P_1(v, t)$ is the marginal probability density of a single "particle" in the mean-field limit, and the collision kernel is given by $Q(P_t, P_t)(v) = Q^+(P_t, P_t)(v) - P_t(v)$, with

$$Q^+(P_t, P_t)(v) := \text{Prob}\Big\{ V_1 + \sum_i^{V_1} X_i^* + \sum_i^{V_2} X_j = v \Big\},$$

X_i^*, X_i, V_1, V_2 being independent random variables such that $P\{V_1 = v\} = P\{V_2 = v\} = P_t(v)$. Setting $Y_i := X_i^* + 1$, one gets a more transparent expression for the gain part of the collision kernel Q^+, i.e.

$$Q^+(P_t, P_t)(v) = \text{Prob}\Big\{ \sum_{i=1}^{V_1} Y_i + \sum_{i=1}^{V_2} X_i = v \Big\}. \tag{S4}$$

Moments and their relaxation dynamics

Considering the probability generating function (pgf) of P_t, $\hat{P}_t(z) := \sum_v z^v P_t(v)$, equation (S3) becomes

$$\frac{\partial}{\partial t}\hat{P}_t(z) = \hat{P}_t(\phi_X(z))\hat{P}_t(\phi_Y(z)) - \hat{P}_t(z) \qquad z \in (0,1) \tag{S5}$$

where $\phi_X(z) = \left\langle z^{X_1} \right\rangle = zp_h + 1 - p_h$ and $\phi_Y(z) = \left\langle z^{Y_1} \right\rangle = z^2 p_d + z(1 - p_d - p_l) + p_l$ are the pgf of X and Y. Deriving (S5) with respect to z one gets

$$\frac{\partial}{\partial t}D_z^{(1)}\hat{P}_t(z) = [D_z^{(1)}\hat{P}_t(\phi_X(z))]\hat{P}_t(\phi_Y(z) + [D_z^{(1)}\hat{P}_t(\phi_Y(z))]\hat{P}_t(\phi_X(z) - D_z^{(1)}\hat{P}_t(z).$$

Recalling that the first derivative of a generating function evaluated in $z = 1$ gives the first moment of the distribution, it follows that $D_z^{(1)}\hat{P}_t(z)|_{z=1} = \sum vP_t(v) =: M_1(t)$, $D_z^{(1)}\phi_X(z)|_{z=1} = \langle X \rangle$ and $D_z^{(1)}\phi_Y(z)|_{z=1} = \langle Y \rangle$. So that the previous equation for $z = 1$ gives

$$\frac{\partial}{\partial t}M_1(t) = (\langle X \rangle + \langle Y \rangle - 1)M_1(t).$$

Now $\langle X \rangle + \langle Y \rangle = p_h + 1 + p_d - p_l = 1$ (since $p_h + p_d = p_l$) and hence $\dot{M}_1(t) = 0$. In conclusion, the mean of the abundance distribution is constant in time,

$$M_1(t) = \sum vP_t(v) = \lambda$$

where λ is the initial mean. Analogously, $D_z^{(2)}\hat{P}_t(z)|_{z=1} = \sum_v (v-1)P_t(v) =: M_2(t)$, $D_z^{(1)}\phi_X(z)|_{z=1} = \langle X(X-1) \rangle$ and $D_z^{(1)}\phi_Y(z)|_{z=1} = \langle Y(Y-1) \rangle$. Consequently, the second derivative (wrt z) of (S5) evaluated in $z = 1$ gives, after some computations,

$$\frac{\partial}{\partial t}M_2(t) + (1 - \langle X \rangle^2 - \langle Y \rangle^2)M_2(t) = [\langle X(X-1) \rangle + \langle Y(Y-1) \rangle]M_1(t) + 2M_1(t)^2 \langle X \rangle \langle Y \rangle .$$

Now, if $Var(P_t)$ denotes the variance of the solution P_t, recalling that $1 = (\langle X \rangle + \langle Y \rangle)^2 = \langle X \rangle^2 + \langle Y \rangle^2 + 2\langle X \rangle \langle Y \rangle$ and that $M_2(t) - M_1(t)^2 = Var(P_t) - M_1(t)$, from the previous equation one obtains

$$\dot{V}ar(P_t) = -\alpha(Var(P_t) - \beta)$$

where

$$\alpha = (1 - \langle X_1 \rangle^2 + \langle Y_1 \rangle^2) = 2p_h(1-p_h)$$
$$\beta = \frac{\lambda[Var(X_1) + Var(Y_1)]}{1 - \langle X_1 \rangle^2 - \langle Y_1 \rangle^2} = \lambda[1 + \frac{p_d}{p_h(1-p_h)}].$$

Solving this equation one obtains

$$Var(P_t) = \beta + [Var(P_0) - \beta]e^{-2p_h(1-p_h)t}. \tag{S6}$$

Similar computations can be performed for higher factorial moments

$$M_k(t) = \sum_v v(v-1)\dots(v-k+1)P_t(v) = D^{(k)}\hat{P}_t(z)\Big|_{z=1}.$$

In point of fact,

$$D^{(k)}[\hat{P}_t(\phi_X(z))\hat{P}_t(\phi_Y(z))]\Big|_{z=1} = M_k(t)(\langle X_1\rangle^k + \langle Y_1\rangle^k) + R_k(t)$$

where $R_k(t)$ is a function of p_h, p_d and of the $M_j(t)$ for $j = 1, \dots, k-1$. Combining this with (S5) one gets a hierarchy of equations

$$\dot{M}_k(t) = -\alpha_k M_k(t) + R_k(t)$$

with $\alpha_k = 1 - \langle X_1\rangle^k - \langle Y_1\rangle^k = 1 - p_h^k - (1-p_h)^k$. Recalling that for $\dot{M}_1(t) = 0$, in principle one can solve recursively the hierarchy obtaining the evolution of any moment. By induction it is easy to see that, for any k, the function R_k is bounded and that $R_k(\infty) = \lim_{t\to+\infty} R_k(t) < +\infty$. Since

$$M_k(t) = e^{-\alpha_k t}M_k(0) + \int_0^t e^{-\alpha(t-s)}R_k(s)ds,$$

one has that the (factorial) moments $M_k(t)$s are bounded in time and

$$\lim_{t\to+\infty} M_k(t) = \frac{R_k(\infty)}{1 - p_h^k - (1-p_h)^k}.$$

Equation (S6) shows that the variance of the mean-field solution converges exponentially fast to the variance of the steady state, with rate set by $p_h(1-p_h)$, in inverse sweeps of $O(N)$ collisions (Fig. S1). In practice, for any finite N, an additional diffusional time scale affects the model. This can be observed in the dynamics of the total abundance of the family $V_{\mathrm{tot}} = \sum_i V_i$, which is subject to a random (multiplicative) addition or deletion at each collision. We can estimate this time scale by assuming a pure diffusion process for the logarithm, with diffusion constant D. In this case, the variance of V_{tot} over model realizations will grow as $Var(V_{\mathrm{tot}}) \sim \exp(Dt)$. For very long times, the variance over realizations can grow larger than the variance of family abundance over genomes. This happens at a crossover time $e^{Dt^*} \sim N\langle V\rangle$. Hence, for times larger than $t^* \sim \log(NV_{\mathrm{tot}})$, the stationary state may be disrupted by this drift process (we measured D to increase with decreasing V_{tot} in simulations, so this should be considered a lower bound). Since t^* grows super-linearly with N, the steady state is always well defined for large enough N. We do not believe that this part of the model phenomenology has a counterpart in the empirical system.

Steady states: Poisson vs overdispersed distributions

If $P_\infty = Q^+(P_\infty, P_\infty)$ is a stationary solution, then (S6) gives

$$Var(P_\infty) = \beta = \frac{\lambda[Var(X_1) + Var(Y_1)]}{1 - \langle X_1 \rangle^2 - \langle Y_1 \rangle^2} = \lambda[1 + \frac{p_d}{p_h(1-p_h)}].$$

This shows that when $p_d = 0$, $Var(P_\infty) = \lambda$, while $Var(P_\infty) > \lambda$ for $p_d > 0$. In this last case, the steady state is characterized by a distribution with larger dispersion than a Poisson with the same average.

When $p_d = 0$, i.e. in absence of duplications, it is easy to show that the stationary solution P_∞ is the Poisson distribution. To see this, recall that the pgf of a Poisson distribution of mean λ is $\hat{P}_\infty(z) = \exp(\lambda(z-1))$. Since, for $p_d = 0$ one gets $\phi_X(z) = zp_h + 1 - p_h$ and $\phi_Y(z) = z(1-p_h) + p_h$, it follows that

$$\hat{Q}^+(\hat{P}_\infty, \hat{P}_\infty)(z) = \exp(\lambda(\phi_X(z) - 1)) \exp(\lambda(\phi_Y(z) - 1)) = \exp(\lambda(z-1)) = \hat{P}_\infty(z).$$

This shows that the steady-state distribution is Poisson, with the same mean as the initial condition. When $p_d > 0$, no closed form for P_∞ is available, although in principle one can recursively determine all the moments. For example, after some computation, one can show that, for a steady state

$$\big\langle (V-\lambda)^3 \big\rangle = \sum (v-\lambda)^3 P_\infty(v) = \lambda\Big(1 + \frac{p_d}{p_h(1-p_h)}\Big(3 + 2\frac{p_d}{p_h}\Big)\Big) \tag{S7}$$

and hence the skewness is

$$skew(P_\infty) = \frac{\langle (V-\lambda)^3 \rangle}{Var(P_\infty)^{\frac{3}{2}}} = \frac{1}{\sqrt{\lambda}} \frac{\Big(1 + \frac{p_d}{p_h(1-p_h)}\Big(3 + 2\frac{p_d}{p_h}\Big)\Big)}{1 + \frac{p_d}{p_h(1-p_h)}}.$$

Finally, using standard techniques of kinetic equations [1] one can prove that for every initial mean $\lambda > 0$, there is a unique fixed point $P_\infty = Q^+(P_\infty, P_\infty)$ with finite variance and mean λ. Moreover, P_t converges to P_∞ whenever P_0 has finite variance. The precise statement is the following: for every $z \in (0, 1]$ and every $t > 0$

$$|\hat{P}_t(z) - \hat{P}_\infty(z)| \leq |z|^2 e^{-2p_h(1-p_h)t} \Big(\sup_{s \in (0,1)} |\hat{P}_0(s) - \hat{P}_\infty(s)|/|s|^2 \Big).$$

Mean-field solution in the limit of vanishing p_d and p_h

Since for $p_d > 0$ no closed form is available for the steady state of the kinetic equation, one can study equation (S5) for $p_d \to 0$ and $p_h \to 0$ provided that $p_d/p_h \to c$. This gives a more tractable equation and leads to an explicit from for the steady state, which is a Negative-Binomial distribution. This kind of asymptotic procedure is reminiscent of the the so-called

"grazing collision" limit for the Boltzmann equation [2, 3]. In kinetic theory, the grazing collision limit is used to derive the Landau-Coulomb equation starting from the Boltzmann equation. Roughly speaking, it consists in taking the limit of collisions giving a small but non-vanishing contribution to the collision integral. In addition to the original Boltzmann equation, the grazing limit procedure has been applied to other kinetic equations (see e.g. [4, 5]).

In the present context, we suppose that $p_d = \epsilon p_d^*$ and $p_h = \epsilon p_h^*$. Expanding $\hat{P}_t(\phi_X(z))$ and $\hat{P}_t(\phi_Y(z))$ for $\epsilon \to 0$, one gets

$$\begin{aligned}\hat{P}_t(\phi_X(z))\hat{P}_t(\phi_Y(z)) - \hat{P}_t(z) &= [\hat{P}_t(1) + \epsilon p_h^*(z-1)\partial_z \hat{P}_t(1) + \epsilon^2 R_1(\epsilon, z)]\cdot \\ &\quad \cdot [\hat{P}_t(z) + \epsilon(p_d^* z^2 + p_d^* + p_h^* - (2p_d^* + p_h^*)z)\partial_z \hat{P}_t(z) + \epsilon^2 R_2(\epsilon, z)] - \hat{P}_t(z) \\ &= \epsilon\lambda(z-1)p_h^*\hat{P}_t(z) + \epsilon(p_d^* z^2 + p_d^* + p_h^* - (2p_d^* + p_h^*)z)\partial_z \hat{P}_t(z) + \epsilon^2 R_3(\epsilon, z)\end{aligned}$$

for suitable remainders $R_i(\epsilon, z)$. One can rescale the time setting $\tau = t\epsilon$ and then consider $\hat{g}_{\tau,\epsilon}(z) = \hat{P}_{\tau/\epsilon}(z)$. Writing t in place of τ and p_d and p_h in place of p_d^* and p_h^* one obtains

$$\partial_t \hat{g}_{t,\epsilon}(z) = \frac{1}{\epsilon}[\epsilon\lambda(z-1)p_h\hat{g}_{t,\epsilon}(z) + \epsilon(p_d z^2 + p_d + p_h - (2p_d + p_h)z)\partial_z \hat{g}_{t,\epsilon}(z) + \epsilon^2 R_3(\epsilon, z)],$$

and, taking the limit for $\epsilon \to 0$, one can write

$$\partial_t \hat{g}_t(z) = \lambda(z-1)p_h\hat{g}_t(z) + (p_d z^2 + p_d + p_h - (2p_d + p_h)z)\partial_z \hat{g}_t(z).$$

If $p_d = 0$, then

$$\partial_t \hat{g}_t(z) = (z-1)p_h[\lambda\hat{g}_t(z) - \partial_z \hat{g}_t(z)].$$

So that the stationary distribution $\hat{g}_\infty$ in the grazing limit satisfies

$$\lambda\hat{g}_\infty(z) = \partial_z \hat{g}_\infty(z)$$

that is $\hat{g}_\infty(z) = \exp(\lambda(z-1))$. Hence, if $p_d = 0$, as for the corresponding kinetic equation, the stationary distribution is a Poisson distribution. Once again, for $p_d = 0$, the only relevant parameter is the initial mean λ. The most interesting case is when $p_d > 0$. Here, since $p_d z^2 + p_d + p_h - (2p_d + p_h)z = p_d(z-1)(z - (p_d + p_h)/p_d)$,

$$\partial_t \hat{g}_t(z) = (z-1)\Big[\lambda p_h \hat{g}_t(z) + p_d\Big(z - \frac{p_d + p_h}{p_d}\Big)\partial_z \hat{g}_t(z)\Big]$$

and the stationary solution satisfies

$$\frac{\lambda p_h}{(p_d + p_h)(1 - \frac{p_d}{p_d + p_h}z)}\hat{g}_\infty(z) = \partial_z \hat{g}_\infty(z).$$

This gives

$$\hat{g}_\infty(z) = \Big(\frac{p_h/(p_d + p_h)}{(1 - \frac{p_d}{p_d + p_h}z)}\Big)^{\frac{\lambda p_h}{p_d}}.$$

In other words, $\hat{g}_\infty$ is the pgf of the negative binomial distribution of mean λ and variance $\lambda(p_d + p_h)/p_h$, that is

$$g_\infty(k) = \binom{k-r-1}{k} \Big(\frac{p_h}{p_d+p_h}\Big)^{\lambda\frac{p_h}{p_d}} \Big(\frac{p_d}{p_d+p_h}\Big)^{k} \quad k = 0, 1, \ldots$$

This expression shows that the steady-state distribution depends in this case on the initial mean abundance λ and on the ratio p_h/p_d.

Model variants.

Model variant accounting for different genome sizes and theoretical justification for the binning procedure.

We now consider a variant of the model that accounts for the different sizes of two interacting genomes in a simplified way. The following argument shows that in this model variant the stationary abundance distributions at a given genome size are unaffected, which can be taken as a heuristic justification of the binning procedure adopted in the data analysis.

One can assume that when the species i and j interact, the abundance of family f in species i – V_i^f – changes in

$$V_i^f(\tau+1) = V_i^f(\tau) + \Lambda_i(V_i^f(\tau)) + H_i(V_j^f(\tau))$$

with $\Lambda_i(V_i^f(\tau)$ as before and

$$H_i(V_j(\tau)) = \sum_{k=1}^{V_j^f(\tau)} X_k^{i,j}(\tau)$$

where $P\{X_k^{i,j}(\tau) = 1\} = p_h N_i(\tau)/N_j(\tau)$ and $P\{X_k^{i,j}(\tau) = 0\} = 1 - p_h N_i(\tau)/N_j(\tau)$, $N_i(\tau)$ and $N_j(\tau)$ being the total size of the genomes i and j, i.e. $N_i = \sum_f V_i^f(\tau)$ and $N_j = \sum_f V_j^f(\tau)$ are the sizes of genome i and j respectively. Note that the mean number of horizontal transfers in a collision (i, j) is

$$\frac{V_j^f(\tau)}{N_j(\tau)} p_h N_i(\tau),$$

which means that the transfer probability is proportional to the abundance of family f in the donor genome j, V_j^f/N_j (which is in turn a proxy for its total size in domains). Clearly, in this model the evolution of the abundance of a family f depends on the evolution of all the other families.

In order to introduce a simplified, but treatable, model, in which the dynamics of each family is independent on the dynamics of the other families, we assume that

$$\left\langle V_i^f \right\rangle \sim \beta_f \left\langle N_i \right\rangle$$

and replace $p_h N_i(\tau)/N_j(\tau)$ with

$$p_h \langle N_i(\tau)\rangle / \langle N_j(\tau)\rangle \sim p_h \left\langle V_i^f(\tau)\right\rangle / \left\langle V_j^f(\tau)\right\rangle.$$

In other words, the assumption states that family size is a good proxy for genome size, for simplicity linear. Empirically, this is justified for metabolic families, but does not happen for other functional categories [6, 7, 8].

With this simplification, one obtains the usual conservation of the mean.

$$\left\langle V_i^f(\tau)\right\rangle = \left\langle V_i^f(0)\right\rangle$$

for every τ and f. Assuming now that the species $i = 1, \dots, N$ are divided in $C_1, \dots, C_M$ bins of equal mean abundance, that is $\left\langle V_i^f(0)\right\rangle = \lambda_k(0)$, for every f in C_k, it is easy to deduce the corresponding Boltzmann-like equation for $P_t(v, k)$, the probability of finding a genome in bin k with abundance v. More precisely one can write

$$\frac{\partial}{\partial t} P_t(v, k) = \sum_{l=1}^{M} [Q^+_{k,l}(P_t(\cdot, k), P_t(\cdot, l))(v) - P_t(v, k)\rho_l(t)] \; , \tag{S8}$$

where $\rho_l(t) = \sum_v P_t(v, l)$ is the fraction of species in size bin l at time t,

$$Q^+_{k,l}(P_t(\cdot, k), P_t(\cdot, l))(v) := \text{Prob}\Big\{ \sum_{i=1}^{V^{(k)}} Y_i + \sum_{i}^{V^{(l)}} X_j^{k,l} = v \Big\} \rho_k \rho_l,$$

$V^{(k)}$ has density $P_t(v, k)/\rho_k$ and $V^{(l)}$ has density $P_t(v, l)/\rho_l$, $P\{X_j^{k,l} = 1\} = 1 - P\{X_j^{k,l} = 1\} = p_h \lambda_k(t)\rho_l(t)/\lambda_l(t)\rho_k(t)$ and $\lambda_l(t) = \sum_v v P_t(v, l)$. Note that $\rho_k(0) = |C_k|/(\sum_m |C_m|)$. Moreover, it is immediate to see that $\rho_k(t) = \rho_k(0) = |C_k|/(\sum_m |C_m|)$ for every t and k and that also the mean abundances are conserved, that is

$$\lambda_k(t) = \sum_v v P_t(v, k) = \lambda_k(0).$$

Note that also in this model, when $p_d = 0$, the Poisson distribution of mean $\lambda_k(0)$, i.e.

$$P_\infty(v, k) = \rho_k e^{-\lambda_k(0)} \frac{\lambda_k(0)^v}{v!},$$

is the stationary solution. To see this, it suffices to use the generating functions, indeed $\hat{P}_\infty(z, k) := \sum_v z^v P_\infty(v, k)$ must satisfy

$$\hat{P}_\infty(\phi_Y(z), k) = \sum_{l=1}^{M} \hat{P}_\infty(\phi_Y(z), k) \hat{P}_\infty(\phi_{X^{kl}}(z), l)$$

where $\phi_{X^{kl}}(z) = \left\langle z^{X_1^{kl}} \right\rangle = zp_h\lambda_k(0)/\lambda_l(0) + 1 - p_h\lambda_k(0)/\lambda_l(0)$ and $\phi_Y(z) = \left\langle z^{Y_1} \right\rangle = z(1-p_l)+p_l$. Now $\hat{P}_\infty(z,k) = \exp\{\lambda_k(0)(z-1)\}$ and hence

$$\begin{aligned}
&\hat{P}_\infty(\phi_Y(z),k)\hat{P}_\infty(\phi_{X^{kl}}(z),l) \\
&\quad = \exp\{\lambda_k(0)[z(1-p_l)+p_l+p_l-1] + \lambda_l(0)[zp_h\lambda_k(0)/\lambda_k(0) + 1 - p_h\lambda_k(0)/\lambda_l(0) - 1]\} \\
&\quad = \exp\{\lambda_k(0)(z-1)\}
\end{aligned}$$

which proves the claim.

Model Variant with Biased Transfers by Evolutionary Distance

We discuss here a model variant accounting for a lower probability of horizontal exchange for increasingly distant genomes, which is reported in empirical data [9]. We implemented this variant in our simulation, by biasing the collisions between genome pairs using information on their phylogenetic distance. Specifically, we simulated a set of genomes corresponding to the 1065 bacteria in our dataset, and for each collision, we adopted the interaction probability between two genomes $\mathrm{prob}(i,j) \propto N^{-1}\exp(-\beta D(i,j))$ for $i \neq j$ ($prob(i,i) = 0$). Here $D(i,j)$ is a phylogenetic distance (we used a distance based on superfamily usage (it is well known that the occurrence patterns of domain families reveals phylogenetic relationships [10]), normalized to lay in the interval $[0,1]$), and β sets the decay of the interaction probability.

Our results (Fig. S8) show that the steady state solution for the abundance histogram remains unvaried by introducing biased gene transfers, until $\beta \simeq 100$. At this range of the bias, each genome in the sample is allowed to interact with 10 or less other genomes only, and the effective rate of horizontal exchange becomes too small to support a steady state. Thus, we can safely conclude that our results are robust with respect to the introduction of a substantial evolutionary bias in horizontal transfers.

References

[1] Bassetti, F., Ladelli, L., and Matthes, D. (2011) Central limit theorem for a class of one-dimensional kinetic equations. *Probability theory and related fields,* **150**(1-2), 77–109.

[2] Villani, C. (1998) On a new class of weak solutions to the spatially homogeneous Boltzmann and Landau equations. *Arch. Rational Mech. Anal.,* **143**(3), 273–307.

[3] Desvillettes, L., Mouhot, C., and Villani, C. (2011) Celebrating Cercignani's conjecture for the Boltzmann equation. *Kinet. Relat. Models,* **4**(1), 277–294.

[4] Pareschi, L. and Toscani, G. (2006) Self-similarity and power-like tails in nonconservative kinetic models. *J. Stat. Phys.,* **124**(2-4), 747–779.

[5] Furioli, G., Pulvirenti, A., Terraneo, E., and Toscani, G. (2012) The grazing collision limit of the inelastic Kac model around a Lévy-type equilibrium. *SIAM J. Math. Anal.,* **44**(2), 827–850.

[6] van Nimwegen, E. (2003) Scaling laws in the functional content of genomes. *Trends in Genetics,* **19**(9), 479 – 484.

[7] Molina, N. and van Nimwegen, E. (2008) The evolution of domain-content in bacterial genomes. *Biology Direct,* **3**(1), 51.

[8] Grilli, J., Bassetti, B., Maslov, S., and Cosentino Lagomarsino, M. (Jan, 2012) Joint scaling laws in functional and evolutionary categories in prokaryotic genomes.. *Nucleic Acids Res,* **40**(2), 530–540.

[9] Andam, C. P. and Gogarten, J. P. (Jul, 2011) Biased gene transfer in microbial evolution.. *Nat Rev Microbiol,* **9**(7), 543–555.

[10] Abeln, S. and Deane, C. M. (2005) Fold usage on genomes and protein fold evolution.. *Proteins,* **60**(4), 690–700.

[11] Podell, S., Gaasterland, T., and Allen, E. (2008) A database of phylogenetically atypical genes in archaeal and bacterial genomes, identified using the DarkHorse algorithm. *BMC bioinformatics,* **9**(1), 419.

[12] Treangen, T. J. and Rocha, E. P. C. (2011) Horizontal transfer, not duplication, drives the expansion of protein families in prokaryotes.. *PLoS Genet,* **7**(1), e1001284.

[13] Debs, C., Wang, M., Caetano-Anolls, G., and Grter, F. (2013) Evolutionary optimization of protein folding.. *PLoS Comput Biol,* **9**(1), e1002861.

Supplementary Figures

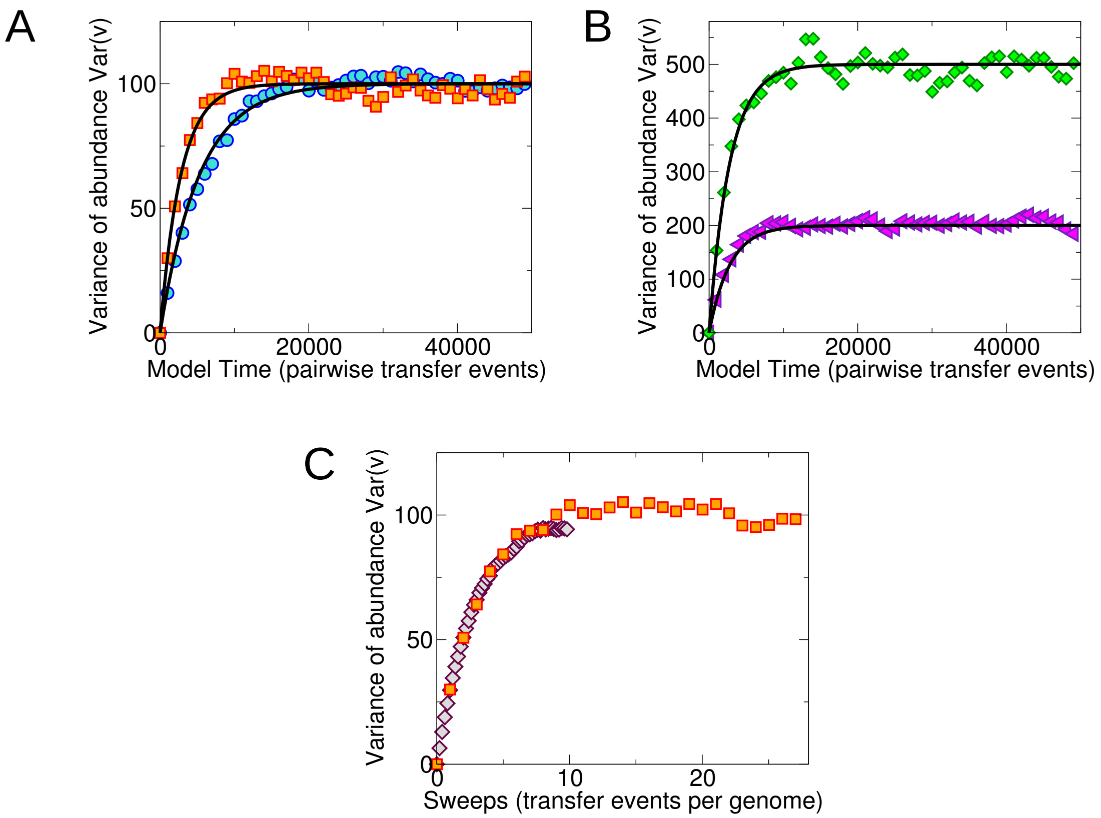

Supplementary Figure S1: The relaxation time to the stationary state is is set by p_h. The plots evaluate the number of iterations in a simulation (corresponding to ”collisional” events of horizontal gene exchange, see Eq. S1) to reach the stationary state from the dynamics of the variance of the family abundance histograms, which we can estimate analytically from the mean-field equations (Eq. (S3)). In the simulations, the initial condition is chosen with zero variance (equal abundance of the family in all genomes). (A) For $p_d = 0$, p_h sets the relaxation scale (see Eq. (S6)), squares correspond to $p_h = 0.1$, circles to $p_h = 0.05$. Simulations are carried out for 1000 genomes. (B) Setting $p_d > 0$ affects the steady state but not the relaxation time. In these simulations $p_h = 0.1$; triangles correspond to $p_d = 0.09$, and diamonds to $p_d = 0.36$. Simulations are carried out for 1000 genomes. (C) The natural time scale of the model are ”collisions” per genome. The plot is a comparison of a simulation with 1000 genomes (squares) and 10000 genomes (diamonds) for equal parameters ($p_d = 0$, $p_h = 0.1$). The time is rescaled by the number of genomes which makes the two plots collapse. In all plots, solid lines are mean-field analytical predictions.

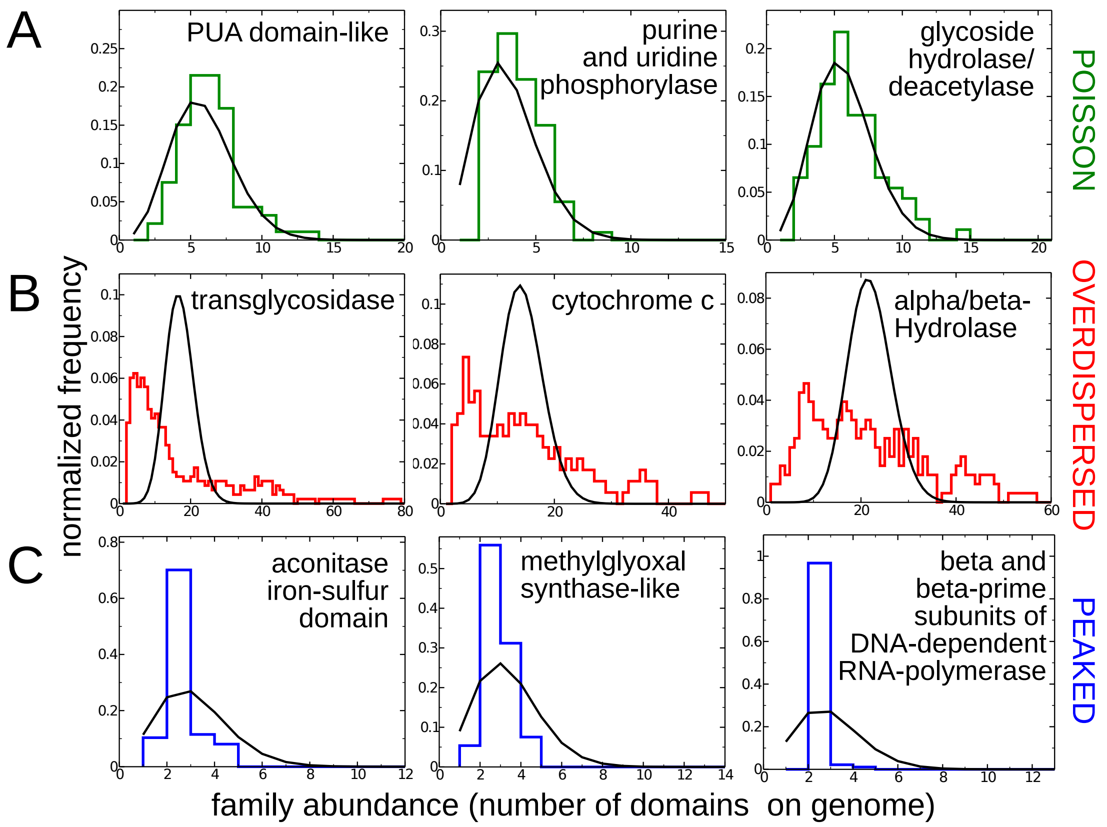

Supplementary Figure S2: Examples of the three main behaviors existing for the family abundance profiles, shown by the insets. As in Fig. 2 of the main text, each panel compares the empirical family abundance histogram (steps) of a given SCOP superfamily domain with the reference Poisson distribution with equal mean (black lines). The plots refer to a group of 93 genomes binned by size (measured in superfamily domains) ranging from 2600 to 3000. "Poisson-like" family profiles (A) correspond to the Poisson expectation, while "overdispersed" (B) and "peaked" (C) abundance profiles are characterized by a larger and lower variance respectively. The typical absolute abundance of the three classes of domain families are different, with families having peaked profiles being less abundant, followed by Poisson-like families and overdispersed ones.

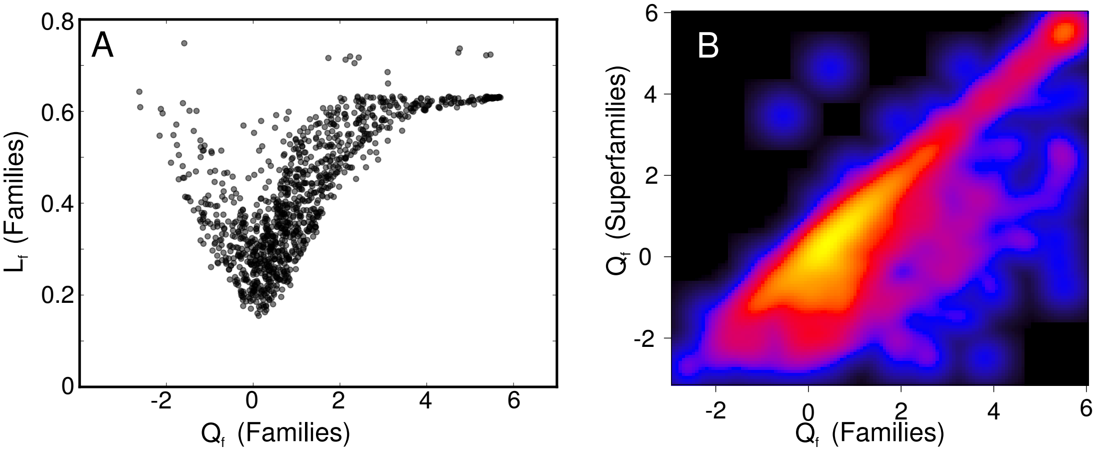

Supplementary Figure S3: Robustness of the abundance profile classification with respect to domain taxonomy. (A) The scatter plot is the same as the one in Fig. 2 of the main text, but realized using SCOP families instead of superfamilies. The fact that it looks unvaried indicates that the classification is robust across domain taxonomy levels, as confirmed by the evident correlation between Q_f computed for a superfamily with the same parameter computed for the corresponding families (B).

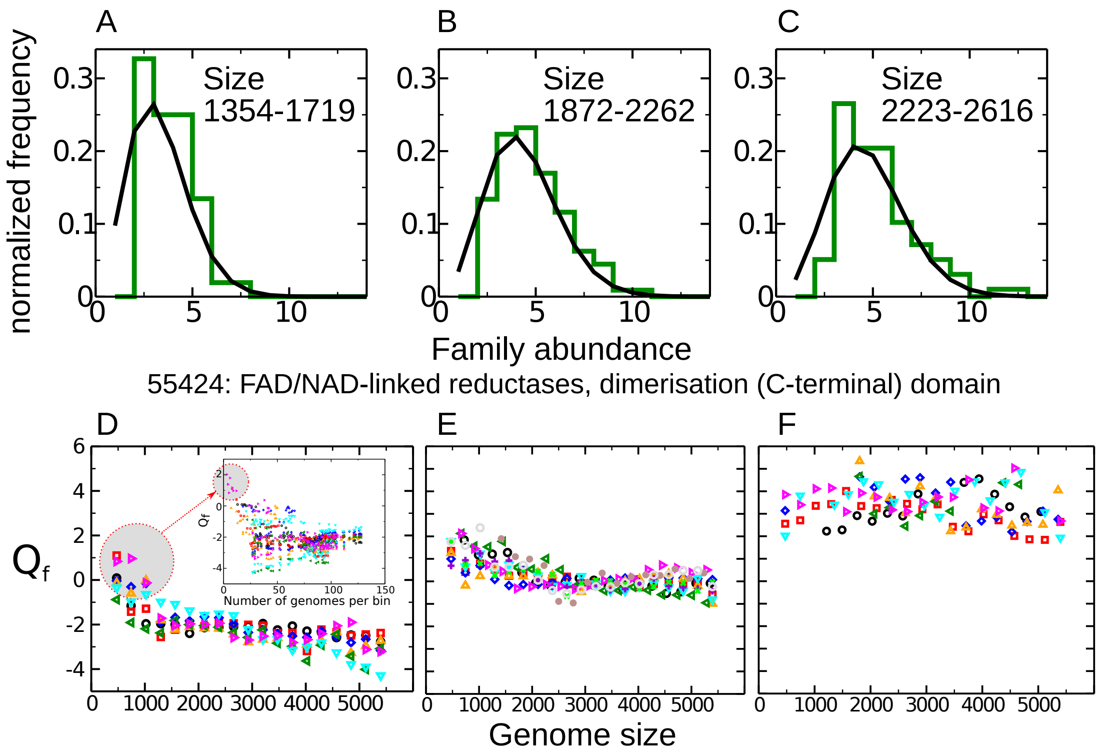

Supplementary Figure S4: The abundance fluctuations of a family do not depend on the genome size. In this example, the abundance histogram of the superfamily with SCOP number 55424 from the SUPERFAMILY database (green stairs) is plotted against the reference Poisson distribution (black line) with equal average for three different bins of genome size (the size intervals, in domains, are indicated in the insets, and increase in panel A,B,C with lexicographic order). While the average class population increases with genome size, the character of the family (in this case Poisson-like) stays the same. The global observables Q_f and L_f are defined in order to account for the robustness of the family behavior over sliding genome-size windows. Panels B,C,D show that the classification operated by Q_f is robust over the bins. The different panels plot the values of Q_f^b for different bins of genome size for families whose abindance profiles are are classified as overdispersed (D) Poisson-like (E) and peaked (F). The inset in panel D shows that the slight trend for small genome size is due to insufficient sampling (few genomes in the bin).

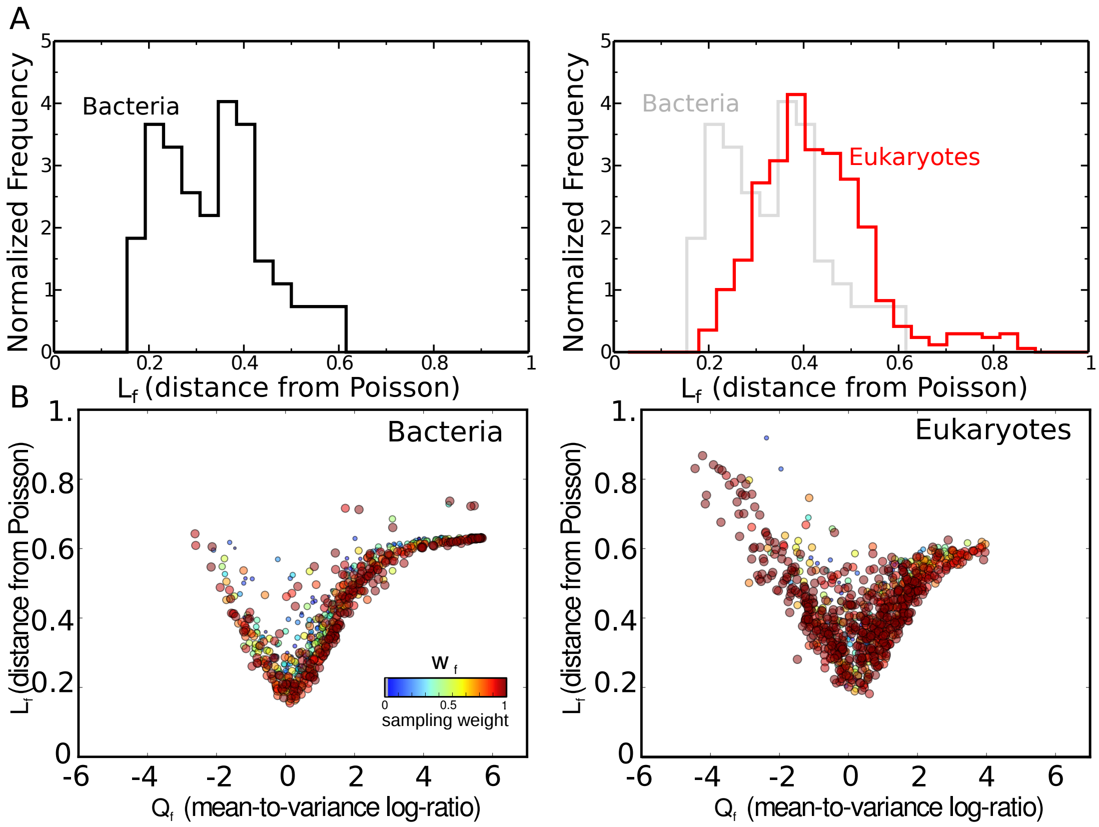

Supplementary Figure S5: Family abundance fluctuations for eukaryotic genomes. (A) Histograms of L_f (weigthed distance from a Poisson distribution) in Bacteria and Eukaryotes. The left panel shows the histogram over families of L_f in Bacteria. The right panel is a comparison between the histograms in Eukaryotes (red line) and Bacteria (gray line). The histogram of Bacteria is be bimodal, suggesting the presence of two main separate kinds of abundance profile. The histogram of Eukaryotes is centered at higher values of L_f and does not show the bimodality. This indicates that families with clear Poisson-like abundance profile families are not as clearly identifiable in Eukaryotes as they are in Bacteria. Both histograms were computed considering well-sampled ($w_f > 0.99$) and non-peaked families ($Q_f < 2$). (B) Comparison of L_f vs Q_f scatterplots in Bacteria and Eukaryotes. Bacteria (left panel) show a clearer V-shape, which becomes more marked as w_f increases. Eukaryotes (right panel) show a less clear relation, which does not improve as sampling (w_f) increases. This fact suggests that in Eukaryotes families with $Q_f \sim 0$ are less prone to actually show consistent Poisson-like abundance profiles.

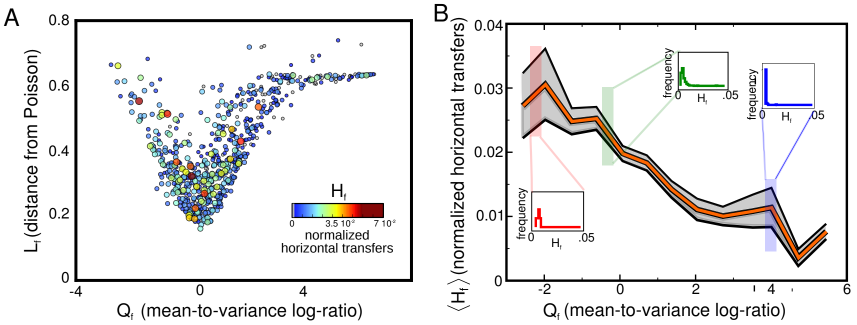

Supplementary Figure S6: Independent estimates of horizontal transfers within families are in line with the model prediction from the family abundance profiles. Panels A and B are identical to Fig. 4B of the main text, but using HGT data from the DarkHorse database [11] (A) $Q_f - L_f$ scatter plot (Fig. 2 of the main text), with color and size of each point corresponding to the parameter H_f (average fraction of horizontally transferred domains in that family estimated using data from the DarkHorse database). Points with $H_f = 0$ are in grey. Compatibly with the expectation of the model, many horizontal transfers are found for Poisson-like families, i.e. towards the minimum of this plot. For $Q_f > 0$ (peaked) families tend to have null H_f. (B) Average of H_f over classes for bins of Q_f. H_f increases with decreasing Q_f, indicating that an increasing number of transfer events are found for families with Poisson-like and overdispersed abundance profiles, compatibly with the model expectations. The insets show the histograms of H_f for the region connected to them.

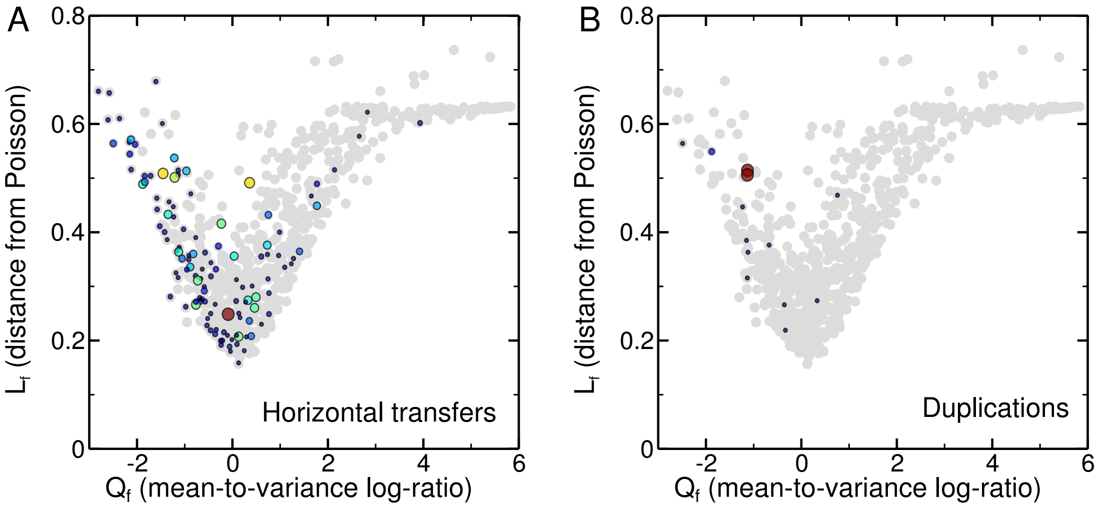

Supplementary Figure S7: Comparison of abundance profiles with an independent evaluation of horizontal transfers and duplications. We considered the data set from Treangen and Rocha [12], who estimated the relative contributions of horizontal transfer and duplication to gene family expansion in a set of closely related complete genomes, and compared them with our classification in terms of family abundance profiles. As in Fig. 4A of the main text, points of the $Q_f - L_f$ scatter plot are colored by average fractions of horizontal transfers (H_f, left panel) and duplications (right panel). Compatibly with the model expectations, colored points are more abundant in the regions where Q_f is negative or close to zero. Grey points have insufficient statistics.

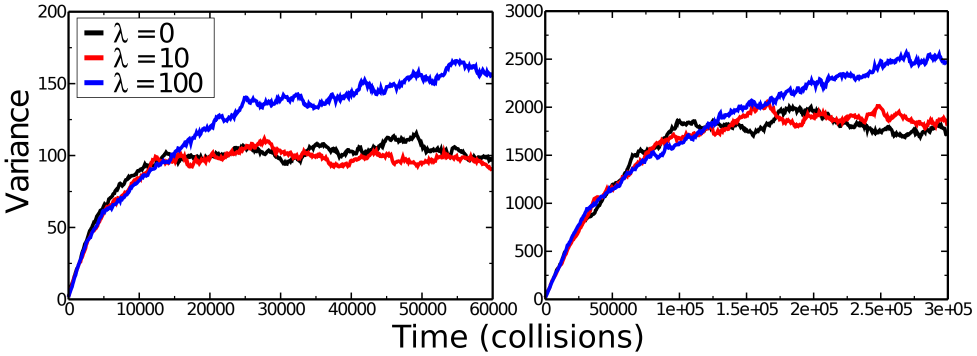

Supplementary Figure S8: A model variant where collisions are biased by evolutionary distance yields unvaried results. We simulated $N = 1065$ genomes biasing their collision probability according to their evolutionary distance. The parameter β sets the strength of the bias, $\beta = 0$ corresponding to the original model without bias. In all the simulations the initial abundance is set to 100, and the initial variance to zero. In the graphs, the variance of family abundance is plotted versus model time (in collisions) for the parameters $p_d = 0.0$, $p_h = 0.05$ (left panel) and $p_d = 0.09, /, p_h = 0.005$ (right panel), and different values of β. The steady-state solution remains unvaried until $\beta \simeq 100$, when interactions are limited to a very a small fraction of genomes.

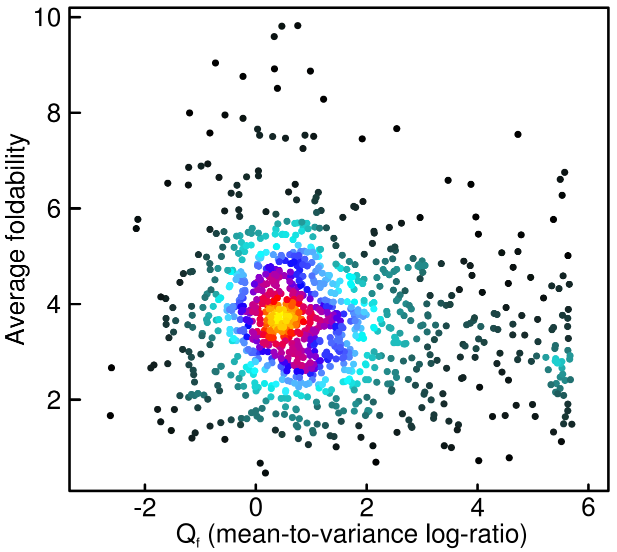

Supplementary Figure S9: Foldability and abundance fluctuations are not correlated. We used data of size-corrected contact order (SMCO) of SCOP domains computed in ref. [13] relating them to the order parameter Q_f of the corresponding families, which measures the abundance fluctuations. Contact order is usually considered a proxy for foldability. The scatter-plot (colored by point density) indicates that foldability is not the main explanation for family abundance fluctuations.

Supplementary Tables

Supplementary Table S1: Relation of the abundance profile of a family with its biological function. The table reports the counts for the larger functional categories of domain superfamilies divided according to their abundance profile histograms following thresholds on Q_f (see main text). P-values for Fisher's exact tests are reported in parenthesis when significant (P< 0.01), in green for overrepresentation and in red for underrepresentation. This table considers a classification based on the abundance fluctuations of all the 1530 superfamilies in the dataset, without filters on the sampling.

	Total 1530	Poisson 277	Overdispersed 193	Peaked 756	Zero Variance 304
General	83	11	15	39	18
Information	173	14 ($7.2 \cdot 10^{-5}$)	8 ($1.8 \cdot 10^{-4}$)	128 ($2.6 \cdot 10^{-12}$)	23 ($1.2 \cdot 10^{-2}$)
Metabolism	513	143 ($4.4 \cdot 10^{-12}$)	72	247	51 ($4.1 \cdot 10^{-13}$)
Processes.EC	59	6	16 ($1.7 \cdot 10^{-3}$)	17 ($8.6 \cdot 10^{-4}$)	20 ($7.0 \cdot 10^{-3}$)
Processes.IC	179	35	32 ($1.9 \cdot 10^{-2}$)	76 ($2.9 \cdot 10^{-2}$)	36
Regulation	142	25	27 ($1.4 \cdot 10^{-2}$)	50 ($2.4 \cdot 10^{-4}$)	40 ($7.9 \cdot 10^{-3}$)
Other	173	23 ($4.7 \cdot 10^{-2}$)	15	86	49 ($2.8 \cdot 10^{-3}$)
N.A	208	20 ($2.1 \cdot 10^{-4}$)	8 ($4.2 \cdot 10^{-6}$)	113	67 ($3.6 \cdot 10^{-6}$)

Supplementary Table S2: Relation of the abundance profile of a family with its biological function. The table reports the counts for the finer functional categories of domain superfamilies divided according to their abundance profile histograms following thresholds on Q_f (see main text). P-values for Fisher's exact tests are reported in parenthesis when significant (P< 0.01), in green for overrepresentation and in red for underrepresentation. This table considers only the 701 families below the noise threshold for the family abundance histograms. The results are completely consistent for a classification of all the 1530 superfamilies in the data set.

		Total 701	Poisson 282	Overdispersed 130	Peaked 281	Zero Variance 8
General	Small.molecule.binding	10	5	4	1 ($4.5 \cdot 10^{-2}$)	0
	Ion.binding	2	0	0	2	0
	Lipid/membrane.binding	0	0	0	0	0
	Ligand.binding	1	0	0	1	0
	General	11	5	4	2	0
	Protein.interaction	8	2	2	4	0
	Structural.protein	0	0	0	0	0
Information	Chromatin.structure	1	0	0	1	0
	Translation	71	3 ($\mathbf{3.1 \cdot 10^{-13}}$)	0 ($\mathbf{2.0 \cdot 10^{-7}}$)	66 ($\mathbf{6.2 \cdot 10^{-23}}$)	2
	Transcription	12	3	1	8	0
	DNA.replication/repair	39	15	4	19	1
	RNA.processing	4	0	0	4 ($2.5 \cdot 10^{-2}$)	0
	Nuclear.structure	0	0	0	0	0
Metabolism	Energy	30	14	0 ($\mathbf{1.8 \cdot 10^{-3}}$)	16	0
	Photosynthesis	1	1	0	0	0
	E-.transfer	10	7	3	0 ($\mathbf{5.7 \cdot 10^{-3}}$)	0
	Amino.acids	20	8	0 ($1.6 \cdot 10^{-2}$)	12	0
	Nitrogen	1	1	0	0	0
	Nucleotide	28	11	1 ($2.2 \cdot 10^{-2}$)	15	1
	Carbohydrate	17	9	4	4	0
	Polysaccharide	6	2	4 ($1.3 \cdot 10^{-2}$)	0 ($4.6 \cdot 10^{-2}$)	0
	Storage	0	0	0	0	0
	Coenzyme	40	20	2 ($1.2 \cdot 10^{-2}$)	18	0
	Lipid	5	4	1	0	0
	Cell.envelope	2	1	1	0	0
	Secondary.metabolism	8	4	2	2	0
	Redox	31	19 ($1.3 \cdot 10^{-2}$)	9	3 ($\mathbf{1.5 \cdot 10^{-4}}$)	0
	Transferases	20	9	5	6	0
	Other.enzymes	96	47 ($3.9 \cdot 10^{-2}$)	19	30 ($3.6 \cdot 10^{-2}$)	0
Processes.EC	Cell.adhesion	6	1	5 ($\mathbf{1.1 \cdot 10^{-3}}$)	0 ($4.6 \cdot 10^{-2}$)	0
	Immune.response	0	0	0	0	0
	Blood.clotting	0	0	0	0	0
	Toxins/defense	3	3	0	0	0
Processes.IC	Cell.cycle,.Apoptosis	4	1	0	3	0
	Phospholipid	1	1	0	0	0
	Cell.motility	6	4	0	2	0
	Trafficking/secretion	1	0	0	1	0
	Protein.modification	19	4	2	13 ($1.1 \cdot 10^{-2}$)	0
	Proteases	18	7	9 ($\mathbf{2.2 \cdot 10^{-3}}$)	2 ($\mathbf{7.5 \cdot 10^{-3}}$)	0
	Ion	19	10	5	4	0
	Transport	24	9	10 ($\mathbf{6.4 \cdot 10^{-3}}$)	5 ($3.7 \cdot 10^{-2}$)	0
Regulation	RNA.binding	8	4	0	4	0
	DNA-binding	22	6	12 ($\mathbf{1.2 \cdot 10^{-4}}$)	3 ($\mathbf{6.7 \cdot 10^{-3}}$)	1
	Kinases/phosphatases	8	7 ($\mathbf{8.5 \cdot 10^{-3}}$)	1	0 ($1.6 \cdot 10^{-2}$)	0
	Signal.transduction	16	5	8 ($\mathbf{3.9 \cdot 10^{-3}}$)	2 ($1.7 \cdot 10^{-2}$)	1
	Other.regulatory.function	7	3	1	3	0
	Receptor.activity	2	1	1	0	0
Other	Unknown.function	42	16	8	16	2
	Viral.proteins	1	1	0	0	0
N.A	not.annotated	20	9	2	9	0
